# Nonlinear Contributions of NO_*x*_ and Volatile Chemical Products to Air Pollution and the Associated Acute Premature Mortality

**DOI:** 10.1021/acsestair.5c00415

**Published:** 2026-05-07

**Authors:** Jiachen Liu, Shannon L. Capps

**Affiliations:** Department of Civil, Architectural & Environmental Engineering, 6527 Drexel University, 3141 Market Street, Philadelphia, Pennsylvania 19104, United States

**Keywords:** CMAQ, sensitivity analysis, volatile chemical products, NO_*x*_, chemical transport models, ground-level ozone, PM_2.5_, premature mortality

## Abstract

Ambient ground-level ozone (O_3_) and fine particulate matter (PM_2.5_) contribute to adverse health impacts. Among controllable emission precursors, anthropogenic nitrogen oxides (NO_*x*_ = NO + NO_2_) and volatile chemical products (VCP) are contributors to O_3_ and PM_2.5_ formation across the continental United States. Accurately assessing the relative contributions of pollution precursors to pollutant formation is crucial for prioritizing emission reduction strategies. Because of the complex formation pathways, the relationship between pollutants and emissions can be highly nonlinear. Traditional finite-difference approaches often fail to fully capture these nonlinearities. In this work, we leverage CMAQ-hyd, an augmented version of the Community Multiscale Air Quality Model (CMAQ v5.3.2), to perform higher-order sensitivity analysis and quantify both linear and nonlinear impacts of NO_*x*_ and VCP on O_3_ and PM_2.5_ formation. Results reveal the impact on acute premature mortality of reducing these precursor emissions. In densely populated cities, a domain-wide reduction in NO_*x*_ emissions could produce significantly more population-weighted O_3_ exposure and associated acute health disbenefits. The reduction of VCP emissions generally leads to reduction in both pollutants, suggesting more opportunities for strategic management especially in densely populated cities. By providing sensitivities, CMAQ-hyd offers a robust framework for evaluating pollutant formation pathways and assessing the health implications of various emission scenarios.

## Graphical abstract

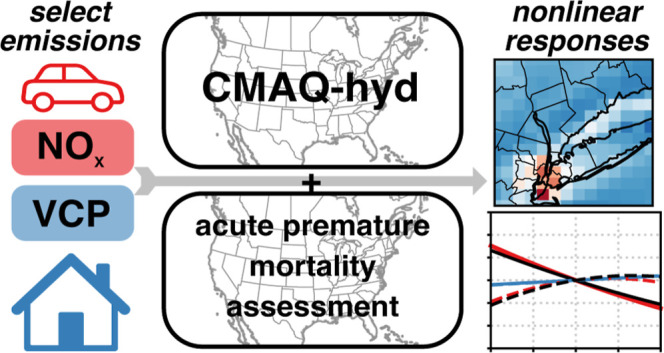

## 1 Introduction

Ambient air pollution from ground-level ozone (O_3_) and fine particulate matter (PM_2.5_) has long been recognized as one of the major environmental threats to public health. The Global Burden of Disease study in 2021 concludes that ambient O_3_ and PM_2.5_ pollution lead to 0.49 million^1^ and 4.72 million^2^ deaths globally, respectively. Understanding how meteorology, precursor emissions, and other factors govern pollutant formation is essential to reducing ambient concentrations and their adverse health impacts. Importantly, sensitivity analysis demonstrates that even small variations in precursor emissions can influence O_3_ production regimes.^3,4^ Furthermore, given the substantial mortality burden attributable to O_3_ and PM_2.5_, these incremental shifts can translate to significant changes in acute premature mortality, as recent epidemiological studies suggests that these pollutants have supra-linear concentration–response functions with no discernible safe threshold.^5,6^

Although regulatory efforts in the U.S. have led to notable air quality improvements, the decline in ground-level O_3_ concentrations has been only 18% from 1990 to 2023, highlighting the difficulty of O_3_ concentration abatement strategies.^7^ In contrast, the annual PM_2.5_ concentration in the US has declined by 37% from 2000 to 2023, mainly driven by improved combustion technologies and tighter emission controls in various sectors.^7^ However, with the new standard of 9 μg/m^3^ annual ambient PM_2.5_ being implemented by the U.S. Environmental Protection Agency (USEPA),^8^ reducing emissions from unconventional sources, including volatile chemical products (VCP), has become vital.

VCP, which include personal care products, surface cleaners, coatings, and paints, can contribute to both ground-level O_3_ and PM_2.5_ formation, particularly in densely populated urban centers.^9–11^ Recent studies have mainly focused on understanding the quantity and speciation of these volatile organic compound (VOC) emissions. McDonald et al. suggested that VCP now could contribute up to half of the total VOC gas phase emissions in industrialized cities in developed nations due to significant reductions of VOC emissions from vehicle tailpipes.^9^ Seltzer et al. developed an emission inventory named VCPy, which supported the conclusion that VCPs could be contributing 50% of the reactive organic carbon emissions in cities.^12^ These VCP can contribute both to PM_2.5_ by forming secondary organic aerosols (SOA) and to O_3_ formation through reactions with NO_*x*_. Both modeling^10,11,13,14^ and observational^15–17^ approaches have helped researchers and policymakers quantify the impact of VCP on PM_2.5_ and O_3_ formation potentials. For instance, Pennington et al. implemented a chemical mechanism for better representation of both oxygenated and nonoxygenated intermediate volatility organic compounds from VCP. By incorporating their new mechanism in the Community Multiscale Air Quality Model version 5.3.2 (CMAQv5.3.2), they found that VCP contribute to noontime SOA, closing the gaps between model predictions and observation in Los Angeles in 2010, and that VCP contribute up to half of the anthropogenic SOA in Los Angeles.^14^ Another study found that the maximum 8 h average (MDA8) O_3_ concentrations are sensitive to anthropogenic VOCs from VCP, even when abundant biogenic VOC sources exist.^11^ A recent study identified optimal traffic-to-VCP emission reduction ratios for mitigating urban O_3_, offering valuable insights for designing effective control strategies.^18^

While the contributions of VCPs to PM_2.5_ are substantial, their impact on O_3_ presents a complex challenge due to the nonlinear dependence on precursor ratios. O_3_ formation is governed by chemical regimes defined by the relative abundance of NO_*x*_ and VOCs.^19^ In densely populated urban environments, O_3_ production is usually characterized as VOC-limited, implying that reductions in VOC emissions could lead to greater O_3_ benefits than reductions in NO_*x*_. In fact, in these VOC-limited schemes, reducing NO_*x*_ emissions could inadvertently lead to an increase in O_3_ concentrations by eliminating the consumption of O_3_ by NO (O_3_ + NO → NO_2_ + O_2_). This phenomenon is often referred to as the “O_3_ weekend effect”, which has been observed in different studies across the globe especially in densely populated urban areas.^20–24^ As the anthropogenic VOC composition in cities shifts toward VCP dominance,^9^ the specific role of VCPs in driving these chemical regimes remains less explored.

While the aforementioned VCP modeling efforts provided the necessary framework for quantifying total emission burdens, most of the evaluations have been done on bulk reduction of emissions. Some of the modeling studies performed sensitivity analysis to understand the extent of the contribution from VCPs, but most of them used “zero-out” finite-difference sensitivity experiments: eliminating VCP emissions from the model and assessing the difference between the modeled concentrations with and without the eliminated emissions.^10,13,14^ While useful for understanding the maximum potential impact, such an approach does not reflect realistic emission reduction scenarios, as large-scale VCP reductions cannot be implemented instantaneously. More detailed analyses of the marginal impacts of incremental VCP reductions are needed to support effective and practical policy design. Furthermore, the chemical coupling between VCPs and other precursors, particularly NO_*x*_, represents cross-sensitivities that simple bulk reductions fail to capture. Quantifying this cross-sensitivity is critical because the efficacy of VCP abatement is not static. Rather, it is dynamically dependent on the concurrent NO_*x*_ burden. Understanding these interactions is critical for identifying potential cobenefits or counteractive effects on pollutant formation that arise in complex multipollutant abatement strategies.

Previous modeling studies^10,11,13,14^ in the literature on air pollutant formation from VCP primarily examined the total concentration and impacts on acute premature mortality. In this manuscript, we highlight the marginal impact of VCP, anthropogenic NO_*x*_, and their interaction through sensitivity analysis with CMAQ-hyd, an augmented version of CMAQv5.3.2, which can compute first- and second-order sensitivities of pollutant concentrations with respect to emissions. We further link these sensitivity results to estimates of marginal health benefits and disbenefits, explicitly accounting for nonlinearities in atmospheric chemistry (e.g., O_3_ chemistry and secondary aerosol formation), atmospheric transport, and deposition processes. This approach provides a more precise and policy-relevant understanding of how targeted precursor reductions affect air quality and public health in complex urban environments.

## 2 Methods

### 2.1 CMAQ and CMAQ-hyd Simulations

CMAQ-hyd^25^ is an augmented version of CMAQv5.3.2,^26^ capable of calculating seminormalized first- and second-order partial derivatives of output concentrations with respect to select input emissions in CMAQ. The augmented model uses the mathematical definition of hyperdual numbers to eliminate the higher-than-second order terms in the Taylor expansion.^27^ Detailed discussion and evaluation of CMAQ-hyd can be found in our previous work.^25^ The numerically exact derivative calculation using hyperdual numbers and their mathematical construction are documented by Fike and Alonso.^27^ In addition to concentration outputs from CMAQ, additional instantaneous and hourly averaged sensitivity information is generated and stored in new output files, using the same metadata structure as the corresponding concentration files.

CMAQ-hyd runs were conducted for two one-week periods: July 16–July 23, 2019 (summer) and January 16–January 23, 2019 (winter), with a two-week spin-up time using CMAQv.5.3.2 in both seasons on NSF-supported computing resources.^28^ For each period, three CMAQ-hyd runs were performed: (1) perturbing anthropogenic NO_*x*_ emissions in dual number space to estimate first- and second-order sensitivities to NO_*x*_; (2) perturbing VCP emissions in dual number space to quantify sensitivities to VCP; and (3) simultaneously perturbing both NO_*x*_ and VCP emissions (in separate dual number spaces) to calculate cross-sensitivities. Details of emission perturbation in the hyperdual space are provided in Liu et al.^25^

Meteorological and emission inputs were obtained from the EPA’s Air Quality TimE Series Project (EQUATES).^29^ The modeling domain (12US1) has 12 km × 12 km horizontal resolution, with 35 vertical layers, 299 rows, and 459 columns, covering the entire continental United States and parts of Canada and Mexico. The full modeling domain, as well as two zoomed-in regions, New York City (NYC) and Greater Los Angeles (LA), are shown in Figure S1. The meteorological inputs are calculated with the Weather Research and Forecasting model version 4.1.1,^30^ and the chemical mechanism is cb6r3-ae7.^31^ The emission inventories include the most up-to-date representation of VCP emissions across the continental United States (CONUS) through VCPy.^12^ A detailed description of the new emission inventories and meteorological inputs can be found in the work by Foley et al.^29^ The EQUATES emission inventories include premerged CMAQ-ready emission files which include different anthropogenic sources including the VCP emissions. To separate the VCP emissions from other gridded anthropogenic emissions, we retrieved the CMAQ-ready VCP emissions file for representative dates across the modeling period, and we created new gridded emissions files without VCP emissions and a set of new VCP emission files. By performing the emission file separation, we were able to perturb the VCP emissions without perturbing emissions from other sources. To ensure numerical stability in the CMAQ-hyd simulations, sensitivity calculations were selectively applied to specific modules within CMAQ. This targeted implementation is consistent with the approach adopted by higher-order decoupled direct method in three dimensions in CMAQ (CMAQ-HDDM), another augmented version of CMAQ designed for sensitivity analysis. A detailed discussion of these methodological compromises can be found in the Supporting Information

CMAQ-hyd outputs averaged, hourly first- and second-order seminormalized sensitivities of pollutant concentrations with respect to emission sources. For each simulation period, sensitivities were averaged over the week according to eq 1.

1 $$s_{X|a}^{\left(1\right)}={\overset{®}{\frac{\partial \overset{®}{C_{X,i,j,k,t}\;}}{\partial E_{a}}E_{a}}|}_{t}={\overset{®}{\frac{\partial \overset{®}{C_{X,i,j,k,t}\;}}{\partial \sigma_{a}}}|}_{t}$$

For each cell in the modeling domain, the sensitivities of average concentrations ( $\overset{®}{C_{X,i,j,k,t}\;}$ ) of pollutant *X* at grid position (*i*,*j*,*k*) of every hour of the model run are further averaged over the modeling period. Here, E_*a*_ is the absolute emission rate of species *a*, and σ_*a*_ is a dimensionless scaling factor used to represent a relative perturbation parameter of species *a*. For each individual cell, three separate simulations, as described earlier in this section, are used to calculate the averaged first-order sensitivities of pollutant *X* with respect to emission of species *a*, denoted as *s*_*X*|*a*_^(1)^; the second-order sensitivities of pollutant *X* with respect to emission of species *a*, denoted as *s*_*X*|*a*_^(2)^; and the cross sensitivities of pollutant *X* with respect to emission of species *a* and *b*, denoted as *s*_*X*|*a*,*b*_^(2)^. These sensitivity values allow for accurate estimation of changes in pollutant concentrations, accounting for linear (first-order) and nonlinear (second-order and cross) interactions between emissions and atmospheric processes. For clarity, we omit the coordinate indices (*i*,*j*,*k*) in all subsequent equations.

### 2.2 Preprocessing of Other Data and Calculation of Concentration Changes and Acute Premature Mortality

Using the hourly average first- and second-order sensitivities, we reconstruct the potential average surface concentration changes due to different emissions. Reconstructing the concentration-emission response using the Taylor expansion of one pollutant *X* from emission *a* follows

2 $$\Delta C_{X|a}\approx s_{X|a}^{\left(1\right)}\delta_{a}+\frac{1}{2}s_{X|a}^{\left(2\right)}\delta_{a}^{2}$$

where ΔC_*X*|*a*_ is the change in concentration of pollutant *X* from emission *a*. s_*X*|*a*_^(1)^ and s_*X*|*a*_^(2)^ are the daily averaged first- and second-order sensitivities of pollutant *X* with respect to emission *a*, as described in Section 2.1. δ_*a*_ is the percentage change in emission *a*. This formula captures both the linear and nonlinear contributions of a single precursor to concentration changes. CMAQ-hyd produces instantaneous hourly sensitivities; however, because the emission perturbation δ is time-invariant, temporally averaged sensitivities yield concentration changes equivalent to those obtained by averaging the hourly concentration changes. This follows from the linearity of the averaging operator and the commutativity of differentiation and finite summation. Accordingly, the hourly sensitivities are first aggregated to 24 h daily averages, consistent with the temporal resolution of the concentration–response functions used in the acute premature mortality assessment described below.

The concentration-emission relationship of one pollutant from two different emissions is given by the Taylor expansion

3 $$\begin{array}{l} \Delta C_{X|a,b}\approx s_{X|a}^{\left(1\right)}\delta_{a}+\frac{1}{2}s_{X|a}^{\left(2\right)}\delta_{a}^{2}+s_{X|b}^{\left(1\right)}\delta_{b}\\ +\frac{1}{2}s_{X|b}^{\left(2\right)}\delta_{b}^{2}+s_{X|a,b}^{\left(2\right)}\delta_{a}\delta_{b} \end{array}$$

where ΔC_*X*|*a*,*b*_ is the hourly average change in concentration of pollutant *X* from precursor emission *a* and *b*. s_*X*|*a*_^(1)^ and s_*X*|*b*_^(1)^ are the hourly averaged first-order seminormalized sensitivities of pollutant *X* with respect to precursor emission *a* and *b*, respectively. s_*X*|*a*_^(2)^ and *s*_*X*|*b*_^(2)^ are the hourly averaged second-order seminormalized sensitivities of pollutant *X* with respect to precursor emission *a* and *b*, respectively. *s*_*X*|*a*,*b*_^(2)^ is the cross-sensitivity of pollutant *X* with respect to precursor emission species *a* and *b*. δ_*a*_ and δ_*b*_ are the relative percentage changes in precursor emissions. The first- and second-order impacts from two distinct emission sources as well as their interaction term s_*X*|*a*,*b*_^(2)^ are included in ΔC_*X*|*a*,*b*_. A positive hourly averaged cross-sensitivity implies a synergistic effect between emissions *a* and *b* on pollutant *X*, whereas a negative value indicates that the two emissions have counteracting effects.

The projected concentration changes were then translated into changes in acute premature mortality for each grid cell by

4 $$\Delta Mortality_{X|a,b}\approx PM_{0}\left(1-e^{-\beta \Delta C_{X|a,b}}\right)$$

where ΔMortality_*X*|*a*,*b*_ is the estimated change in mortality from pollutant *X*, resulting from precursor emissions *a* and *b*. P is the population residing in the specific cell at ground level, derived from 2019 U.S. Census Bureau population estimates^32^ using the Environmental Benefits Mapping and Analysis Program–Community Edition (BenMAP–CE).^33^ Census block-level population data were spatially allocated to the 12 km CMAQ modeling grid (12US1). The population data are plotted in Figure S2. m_0_ is the baseline mortality in the cell. β is the concentration–response coefficient, and ΔC_*X*|*a*,*b*_ is the change in pollutant concentration from precursor emission changes. To keep the study design to a reasonable simulation time, we employed the acute 24 h average concentration–response coefficients for O_3_ and PM_2.5_ to assess the acute premature mortality effects commensurate with the week-long modeling period.^34,35^ A detailed evaluation and discussion confirming the representativeness of the selected modeling week is provided in Figures S3–S6. The hourly surface CMAQ-hyd O_3_ and PM_2.5_ concentrations are thus aggregated to 24 h daily averages to maintain consistency with the exposure metric which the concentration–response relationships were derived. Combining eqs 3 and 4, we derive relationships between the change in mortality and the relative percentage change in emissions. Since the concentration-emission relationship considers higher-order sensitivities, the relationship more accurately reflects the impacts of marginal changes in emissions.

To estimate the total change in mortality attributable to pollutant *X* from precursor emissions *a* and *b* within a specific region and season, we aggregate the daily, cell-level results by

5 $${Seasonal\;\Delta Mortality}_{X|a,b}\approx \Delta t\sum_{i}\;\sum_{j}\Delta Mortality_{X|a,b}$$

where Seasonal ΔMortality_*X*|*a*,*b*_ represents aggregated seasonal mortality across all surface grid cells within the modeling domain due to pollutant *X* from precursor emissions of *a* and *b*. ΔMortality_*X*|*a*,*b*_ is the estimated change in mortality of pollutant *X* from precursor emission of *a* and *b* in each day. Indices *i* and *j* refer to the *x*- and *y*-direction of the surface grid, respectively. We only consider surface cells in this study. Δ*t* denotes the number of days in each season: 92 days for summer (June–August, JJA) and 90 days for winter (December–February, DJF).

We also computed the population-weighted concentration change within a specific region (eq 6). Here, Δ*C*_pw,*X*|*a*,*b*_ is the population-weighted concentration of pollutant *X* from emission *a* and *b*. ΔC_*X*|*a*,*b*_ is the change in concentration calculated in eq 3. P represents the population in individual cells at ground level.

6 $$\Delta C_{pw,X|a,b}=\frac{\sum_{i}\;\sum_{j}\Delta C_{X|a,b}P}{\sum_{i}\;\sum_{j}P}$$

Additionally, the contribution from an individual sensitivity term can be computed. For instance, the population-weighted, first-order contribution to pollutant *X* from emission *a* is

7 $$\Delta C_{pw,s_{X|a}^{\left(1\right)}}=\frac{\sum_{i}\;\sum_{j}\Delta C_{s_{X|a}^{\left(1\right)}}P}{\sum_{i}\;\sum_{j}P}$$

where $\Delta C_{pw,s_{X|a}^{\left(1\right)}}$ represents the population-weighted concentration change based on the first-order impacts of pollutant *X* from emission *a*. The term $\Delta C_{s_{X|a}^{\left(1\right)}}$ corresponds to the first component in eq 3, defined as s_*X*|*a*_^(1)^δ_*a*_. Similarly, the contributions from all other components from eq 3 can be computed.

## 3 Results and Discussion

### 3.1 Domain-Wide Impact of Emission Reductions

A 50% domain-wide reduction in NO_*x*_ and VCP emissions affects O_3_ and the associated mortality differently in the summer and winter (Figure 1). Overall, the impacts are primarily driven by NO_*x*_ reductions, while reductions in VCP emissions also contribute to lower O_3_ concentrations across the CONUS, albeit to a lesser extent (Figures S7–S8). During summer, a domain-wide reduction in both precursors generally results in decreased O_3_ concentrations across most of the CONUS (Figure 1a), with notable exceptions in some metropolitan areas (e.g., Los Angeles, CA; Denver, CO). In these cities, the reductions are less pronounced or even reversed. In winter, domain-wide reductions in emissions lead to O_3_ increases in many areas, reflecting the nonlinear and regime-dependent relationship between O_3_ and NO_*x*_. In summer, abundant solar radiation and biogenic VOC emissions^36^ drive rapid photochemical O_3_ production under predominantly NO_*x*_-limited conditions, where NO_*x*_ reductions effectively lower O_3_. In winter, reduced photolysis rates and minimal biogenic VOC emissions result in NO_*x*_-saturated (VOC-limited) conditions over much of the domain. Under these conditions, O_3_ titration by NO dominates the local O_3_ budget, and reductions in NO_*x*_ emissions diminish this titration sink, leading to O_3_ increases, particularly in urban and near-source areas.^37^

**Figure 1:**
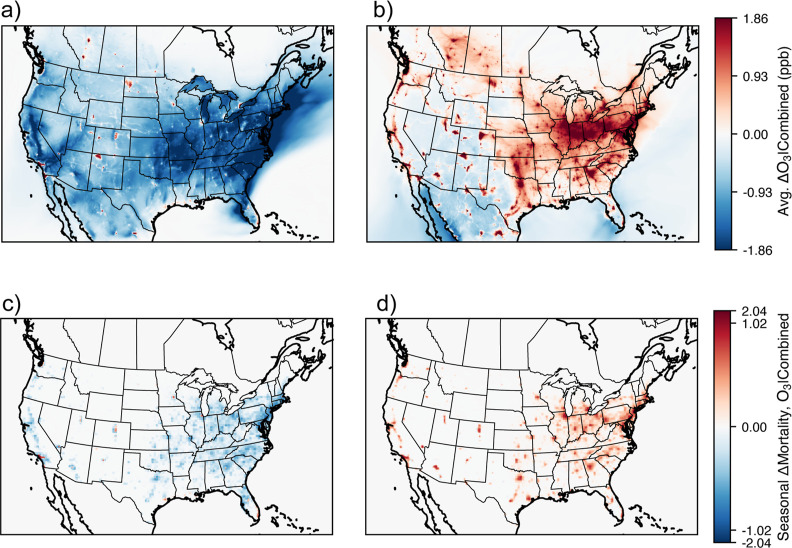
Surface O_3_ concentration changes due to a 50% reduction in domain-wide reduction in NO_*x*_ and VCP emissions in summer (a) and in winter (b). The changes in mortality due to O_3_ in summer (c) and in winter (d). Concentration change panels use a linear, two-slope color scale clipped at the ±98th percentile of the delta-values to avoid plotting the extreme values on the colormap.

Although mitigating VCPs yields reductions in O_3_, the benefits are insufficient to offset the health disbenefits associated with NO_*x*_ reductions in winter (Figure 1c,d). Changes in acute premature mortality are evaluated only within the US portion of the CMAQ modeling domain, although the full modeling domain includes parts of Canada and Mexico. The combined reduction of NO_*x*_ and VCP emissions leads to an estimated decrease of 373.2 (95% confidence interval: −194.0, −552.0) acute premature mortality in summer (Table 1). In contrast, winter reductions result in an increase of 489.1 (95% confidence interval: 254.4, 723.1) O_3_-related deaths, highlighting the potential health disbenefits of domain-wide NO_*x*_ control during colder months. Previous studies^19,38^ have shown that the reduction of NO_*x*_ emissions can increase O_3_ concentrations due to reduced NO titration, suggesting potential adverse effects of emission control strategies. Our study further quantifies the associated health disbenefits of emission control strategies, highlighting the importance of understanding region-specific atmospheric chemistry and developing appropriate policies to protect public health. The individual impacts of 50% domain-wide reduction in NO_*x*_ or VCP on PM_2.5_ across continental United States are listed in Figures S9–S10.

**Table 1:** Domain-wide Total Seasonal Changes in Mortality From 50% Reduction in Emissions^a^

season	emission	pollutant	Δ mortality (95% CI)
summer	NO_*x*_	PM_2.5_	–11.9 [−8.5, −14.8]
		O_3_	–352.9 [−183.4, −522.0]
	VCP	PM_2.5_	–2.7 [−2.0, −3.4]
		O_3_	–28.4 [−14.8, −42.1]
	combined	PM_2.5_	–14.2 [−10.2, −17.7]
		O_3_	–373.2 [−194.0, −552.0]
winter	NO_*x*_	PM_2.5_	–1.14 [−0.82, −1.42]
		O_3_	516.0 [268.3, 762.9]
	VCP	PM_2.5_	–1.6 [−1.1, −2.0]
		O_3_	–26.1 [−13.4, −38.6]
	combined	PM_2.5_	–2.9 [−2.1, −3.6]
		O_3_	489.1 [254.4, 723.1]

a The 95% confidence intervals (CIs) are derived from the uncertainty in the concentration–response coefficient (β).

The acute mortality impacts associated with PM_2.5_ formation from NO_*x*_ and VCP emissions are generally smaller than those linked to O_3_. A 50% reduction in both precursors results in an estimated decrease of 14.2 (95% confidence intervals: −10.2, −17.7) deaths in summer and 2.9 (95% confidence intervals: −2.1, −3.6) deaths in winter. However, several metropolitan areas exhibit increased PM_2.5_-related mortality despite emission reductions, especially during wintertime. This counterintuitive effect may stem from elevated O_3_ concentrations in cities, which increase the atmospheric oxidant burden and promote secondary sulfate formation, thereby slightly increasing PM_2.5_ concentrations and associated acute premature mortality (Figure 2). This effect is also observed and modeled by Shah et al., whose work demonstrated that massive reductions in emissions over the eastern United States produced little change in wintertime PM_2.5._^39^ This dampening effect occurs because emission reductions alleviate oxidant limitations and alter fine particle acidity, thereby increasing the chemical efficiency of secondary sulfate and nitrate formation.^39^ This strong chemical feedback underscores the limitation of simple “zero-out” approaches and highlights the necessity of using advanced sensitivity tools, such as CMAQ-hyd, to accurately capture marginal impacts under shifting chemical regimes. In addition, our results indicate that gas-phase atmospheric chemistry likely governs the net responses of PM_2.5_ and O_3_ to NO_*x*_ and VCP emission reductions in our modeling domain. These outcomes inherently incorporate the indirect effect, such as weakened radical scavenging and photolysis shielding, that can otherwise stimulate O_3_ production as PM_2.5_ concentration decreases in heavily polluted or smoke-impacted regions.^40,41^ Additional mortality-related results with 10% and 25% reductions in both precursors are shown in the Table S1 and S2.

**Figure 2:**
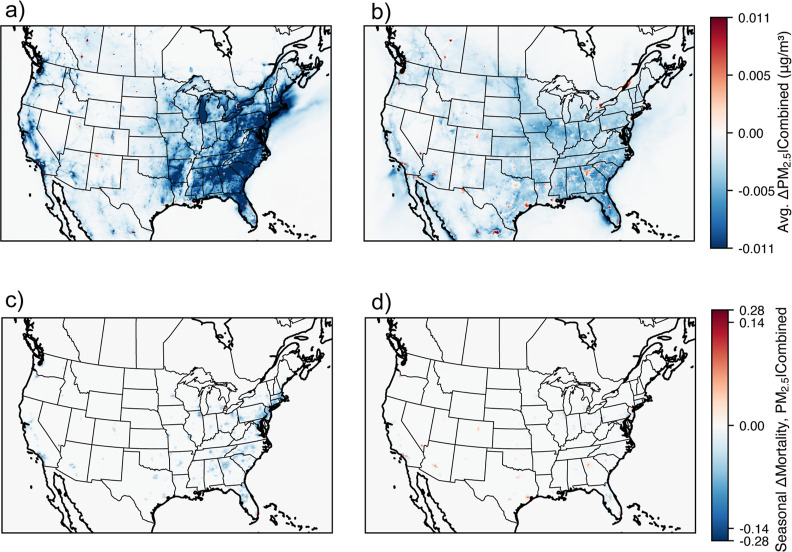
Surface PM_2.5_ concentration changes due to a 50% reduction in domain-wide reduction in NO_*x*_ and VCP emissions in summer (a) and in winter (b). The changes in mortality due to PM_2.5_ in summer (c) and in winter (d). Concentration changes panels use a linear, two-slope color scale clipped at the ±98th percentile of the delta-values to avoid plotting the extreme value on the color bar.

The seasonal changes in mortality resulting from 50% reductions in NO_*x*_, VCP, and their combination, including their respective effects on PM_2.5_ and O_3_ concentrations across CONUS are associated with net decreases in mortality except for the domain-wide NO_*x*_ reduction, which dominates the combined mortality outcome for O_3_ in winter. The 95% confidence intervals (CIs) for each emission–pollutant–mortality relationship are derived from the uncertainty in the concentration–response coefficient, β, reported in the corresponding epidemiological studies. It is important to note that the individual impacts of NO_*x*_ and VCP emissions are calculated using eq 2, whereas the combined impact is derived using eq 3, which accounts for cross-sensitivities. As a result, the combined effect is not a simple sum of the individual contributions but incorporates interactions between the precursors.

The seasonal changes in O_3_-related mortality commensurate with a range of potential changes in NO_*x*_, VCPs, and their combined effects elucidates strategic approaches to emissions controls and demonstrates the value of this sensitivity analysis approach (Figure 3). The influence of NO_*x*_ shows distinct seasonal behavior: in summer, there is a positive relationship between NO_*x*_ emissions and O_3_-related mortality (solid red line), whereas in winter, this relationship becomes negative (dashed red line). This contrast arises because reducing NO_*x*_ emissions can increase O_3_ concentrations in densely populated cities, particularly during winter, due to VOC-limited O_3_ chemistry. In summer, although urban areas exhibit similar effects (Figure 1a), the overall relationship between NO_*x*_ and mortality becomes negative because O_3_ formation outside urban centers remains NO_*x*_-limited, so reducing NO_*x*_ lowers O_3_ concentrations and associated acute premature mortality. On the other hand, reducing VCP emissions consistently decreases O_3_-related mortality, with a much smaller seasonal variation compared to NO_*x*_ though by an order of magnitude less than NO_*x*_ emissions reductions. While O_3_ exhibits strong responses to precursor emissions, the sensitivity of PM_2.5_ demonstrates a weaker response profile (Figure S11). Although the acute premature mortality associated with PM_2.5_ is smaller in magnitude compared to O_3_ in this domain, capturing these sensitivities remains critical for comprehensive policy evaluation.

**Figure 3:**
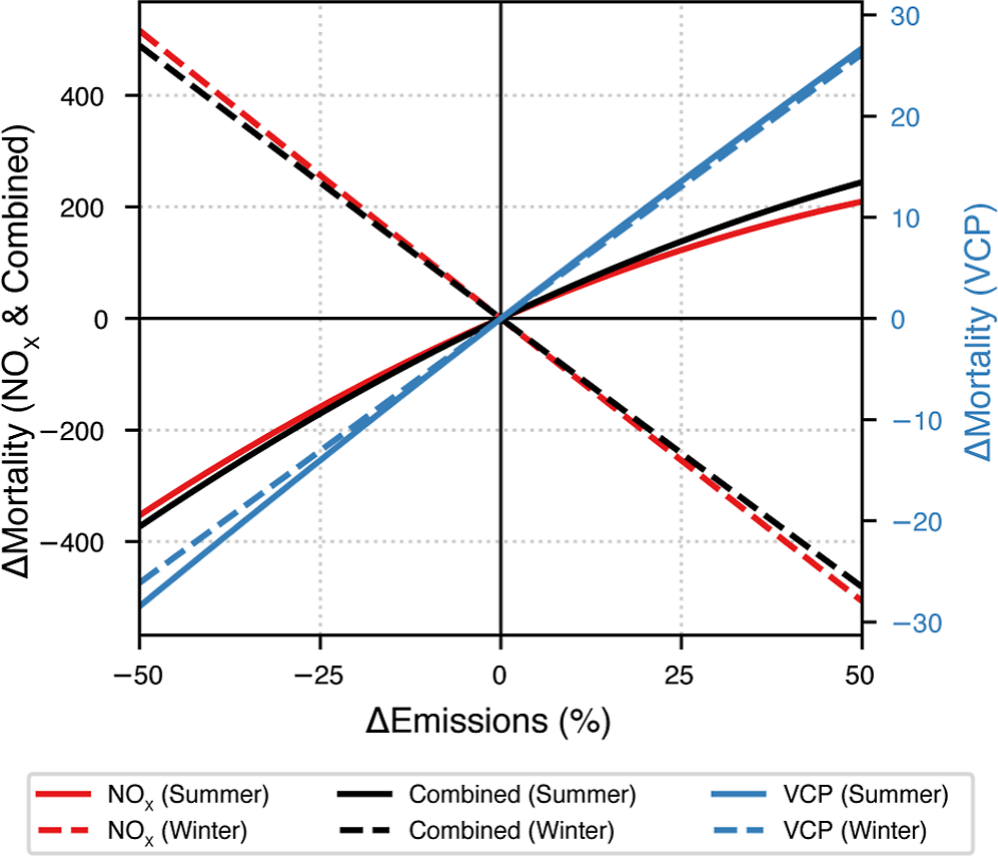
Cumulative changes in mortality from O_3_ due to NO_*x*_ (red), VCP (blue), and combined impact of both (black) in summer (solid lines) and in winter (dashed lines) with the range of change in emissions from −50% to +50%. Two separate *y*-axes are employed to demonstrate the impact of NO_*x*_ and combined impact (black *y*-axis) and the impact of VCP (blue *y*-axis).

Both the impact of NO_*x*_ and VCP on O_3_-related mortality changes display some nonlinearity, underscoring the importance of including the second-order sensitivities when calculating health-related outcomes. The combined effect of NO_*x*_ and VCP emissions closely resembles the NO_*x*_-only response but additionally includes cross-sensitivities between the two precursors. Importantly, the combined impact is not a simple summation of individual contributions, and it captures interactions between precursors. While cross-sensitivities play a relatively minor role in this case, they may become more pronounced in other atmospheric regimes, such as those involving interactions between biogenic isoprene and NO_*x*_. For instance, one previous study found that anthropogenic NO_*x*_ emission reductions lead to decreased O_3_ sensitivities to biogenic isoprene.^42^ Another study explored the synergistic impact of various emission scenarios on O_3_ and PM_2.5_ formation and identified optimal NO_*x*_-to-VOC reduction ratios for different conditions.^43^ The cumulative mortality changes from PM_2.5_ due to NO_*x*_, VCP, and their combined effects are much smaller than those arising from the formation of O_3_ from these precursors (Figure S11). These findings highlight the necessity of accounting for cross-sensitivities when evaluating the joint impacts on acute premature mortality from multiple emission sources. CMAQ-hyd enables precise calculation of these cross-terms, providing a robust tool for evaluating the nonlinear interactions between precursors.

### 3.2 Region-Specific Impact of Emission Reductions

The gradient of average O_3_ concentrations in selected grid cells in NYC (Figure 4a) and in Greater Los Angeles (LA) (Figure 4d) during the summer demonstrates the effect of NO titration in the urban core. In each subfigure, two representative grid cells are highlighted using white outlines: one located within densely populated urban areas (solid white boxes), and the other located more downwind of these areas, outlined in dashed white boxes. The average hourly concentrations for each cell are directly annotated on the figures. In both cities, O_3_ concentrations tend to be lower in the more densely populated core areas compared to the downwind locations, where higher concentrations are observed. To illustrate the regional emission response characteristics and pixel-level variability, Figure 4b,e display the spatial distribution of Δ*O*_3_ resulting from a 50% reduction in both NO_*x*_ and VCP emissions across CONUS. These spatial maps reveal highly heterogeneous responses to a uniform emission reduction strategy, emphasizing the strong spatial gradients in chemical regimes across both urban domains. The pixel-level responses of O_3_ concentration to individual perturbation of NO_*x*_ and VCP emissions are shown in Figures S12.

**Figure 4:**
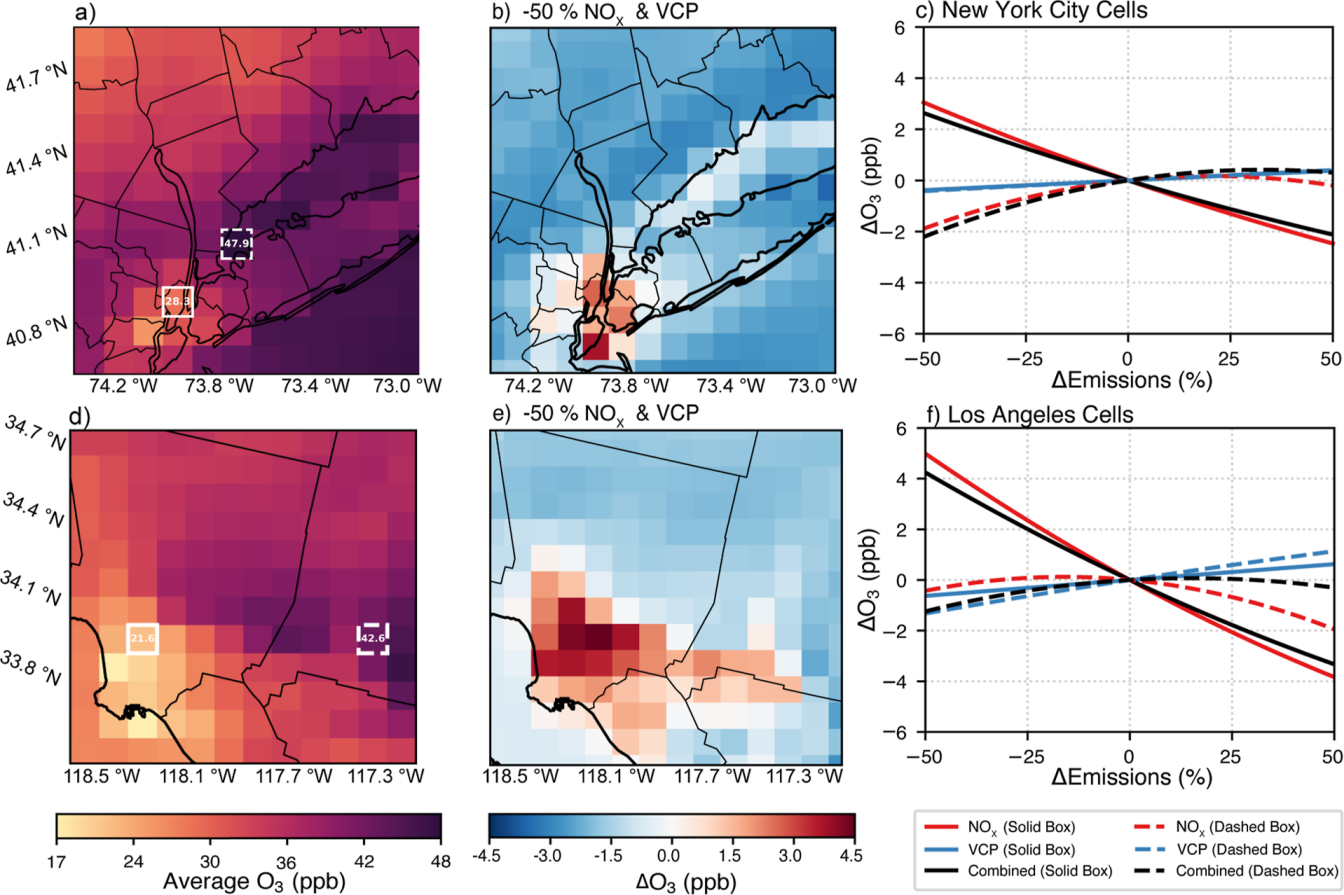
Average O_3_ concentrations in the summer week of the simulation for the grid cells near (a) New York City and (d) Los Angeles (LA) cells, with latitude and longitude ranges specified. The average concentrations for two specific cells are written on the spatial maps in solid boxes and dashed boxes. Panels (b,e) illustrate the spatial distribution of changes in O_3_ concentrations (ΔO_3_) within these domains resulting from a simultaneous 50% reduction in both NO_*x*_ and VCP emissions near New York City (b) and Los Angeles (e). Panels (c,f) represent the calculated concentration-emission relationships of O_3_ to individual domain-wide changes in NO_*x*_ (in red), VCP (in blue), and combined NO_*x*_ and VCP emissions (in black) for New York City and Los Angeles, respectively, calculated by eq 2 for each cell. The box borders on plots (a,d) match the line styles of the concentration–response curves in (c,f). For example, the average O_3_ concentration (28.3 ppb) in the solid box cell in (a) responds to individual changes in domain-wide NO_*x*_ (red solid line in (c)) and VCP (blue solid line in (c)), as well as combined changes (black solid line in (c)).

Figure 4c,f display the response of O_3_ concentrations in these cells to domain-wide reductions in NO_*x*_ and VCP emissions. In the densely populated urban cells (solid lines), NO_*x*_ reductions lead to an increase in O_3_, indicating VOC-limited chemistry. Conversely, in the higher-concentration downwind cells, the response of O_3_ to NO_*x*_ reductions is markedly nonlinear, as indicated by the curvature of the response curves. For instance, in the downwind NYC grid cell (dashed box over Long Island Sound), a reduction in NO_*x*_ emissions leads to a clear decrease in O_3_, while in the analogous LA grid cell, O_3_ initially increases slightly before declining—demonstrating the importance of capturing second-order effects. These findings underscore the limitations of relying solely on first-order sensitivities, which may fail to reflect the true nonlinear behavior of O_3_ formation under varying precursor emission levels. In contrast, the response to VCP emissions appears more linear in both cities, with domain-wide reductions consistently leading to lower O_3_ concentrations. The black solid lines in Figure 4c,f illustrate the O_3_ response to simultaneous reductions in both NO_*x*_ and VCP emissions. These combined impact curves reveal how joint mitigation strategies interact across different local chemical regimes. For example, while combined reductions lessen the severity of the NO_*x*_ disbenefit in the VOC-limited urban cores, the titration effect still initially dominates. Conversely, the downwind receptor cells experience an immediate and sustained decrease in O_3_ under the combined reduction scenario. Overall, Figure 4 illustrates the capability of CMAQ-hyd to compute grid-cell-specific pollutant responses to precursor emissions with machine precision, providing valuable insights into the nonlinear chemical regimes that drive air quality outcomes.

The joint response surfaces of O_3_-related mortality to domain-wide NO_*x*_ and VCP emission changes for NYC and Greater LA during summer and winter (Figure 5) summarize the influences of a range of domain-wide precursor reductions on O_3_, population distributions, and locally variable baseline mortality. The values reflect cumulative percent changes in mortality, calculated using eq 5, across a perturbation range of ±50% for both precursors. Negative values indicate avoided deaths, while positive values reflect increased mortality due to emission changes. In all four panels, the directionality of the contours confirms a VOC-limited chemical regime: mortality increases with NO_*x*_ reductions (leftward movement), while VCP reductions (downward movement) generally lead to decreased mortality. Notably, the magnitude and steepness of the mortality gradients vary by region and season. However, these differences should not be interpreted solely as differences in atmospheric chemical sensitivity. Rather, they also reflect underlying differences in baseline population, mortality rates, and spatial distributions of O_3_ exposure. For example, Greater LA in summer (Figure 5b) shows a large dynamic range in mortality response, but this is in part due to its larger exposed population, larger modeling domain, and higher baseline O_3_ levels compared to NYC in winter (Figure 5c), which exhibits flatter gradients. However, it is important to note that the current analysis is conducted at a relatively coarse spatial resolution, which may not fully capture intraurban variations in pollutant exposure and population vulnerability. As a result, finer-scale modeling with CMAQ-hyd or other augmented models is necessary to better resolve spatial gradients in O_3_ concentrations and accurately assess localized impacts on premature mortality. Moreover, our analysis is based on domain-wide emission perturbations across the CONUS modeling domain, which may not capture the impacts of strictly local precursor reductions. More location-specific pollution mitigation strategies on a higher-resolution modeling domain would also be critical for designing more effective, spatially targeted emission control strategies, especially in urban environments where population density and chemical regimes can vary significantly within a single metropolitan area, as demonstrated in Figure 4. Such improvements would help maximize health benefits and reduce the risk of unintended disbenefits from poorly targeted precursor emission reductions. The joint response surfaces of PM_2.5_-related mortality to domain-wide NO_*x*_ and VCP reductions are shown in Figure S13.

**Figure 5:**
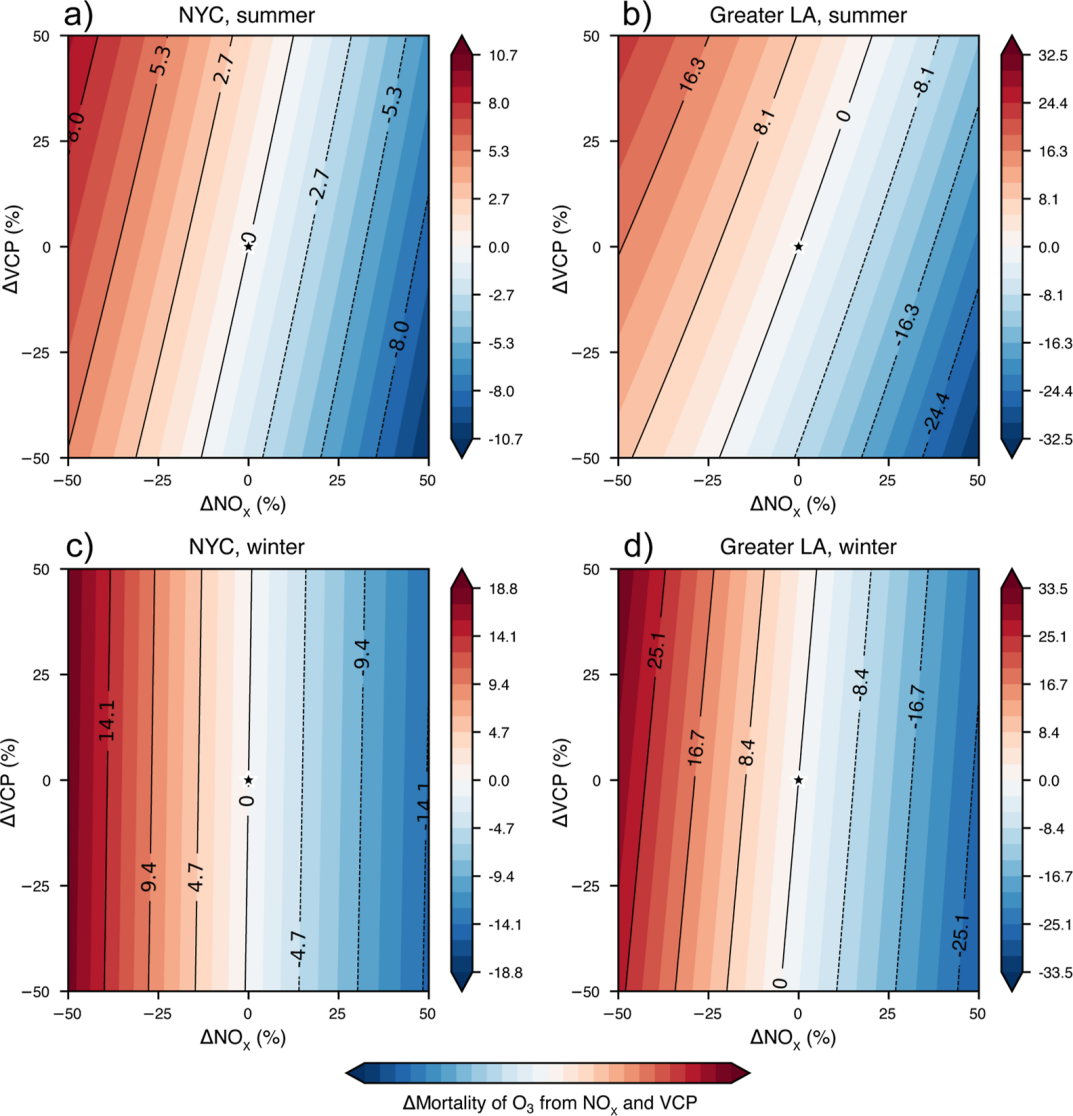
Total O_3_-related mortality changes in response to domain-wide NO_*x*_ and VCP emission perturbations, calculated using eq 5 and aggregated for four urban-region and seasonal scenarios: (a) NYC in summer, (b) Greater LA in summer, (c) NYC in winter, and (d) Greater LA in winter. Emission perturbations for both NO_*x*_ and VCPs range from −50% to +50%. All mortality estimates account for both first-order and cross-sensitivities between precursors.

The population-weighted concentration changes for PM_2.5_ and O_3_ in NYC and Greater LA can be decomposed into the impacts from the five sensitivity terms in eq 3 (Figure 6). Population-weighted concentrations were calculated using eq 7 for a scenario involving a 50% reduction in domain-wide NO_*x*_ and VCP emissions. Here, a positive value indicates that the emission reduction leads to an increase of the population-weighted concentration of the pollutant, and vice versa. Therefore, a positive value indicates the corresponding sensitivity term (e.g., $s_{PM_{2.5}|NO_{x}}^{\left(1\right)}$ ) is negative.

**Figure 6:**
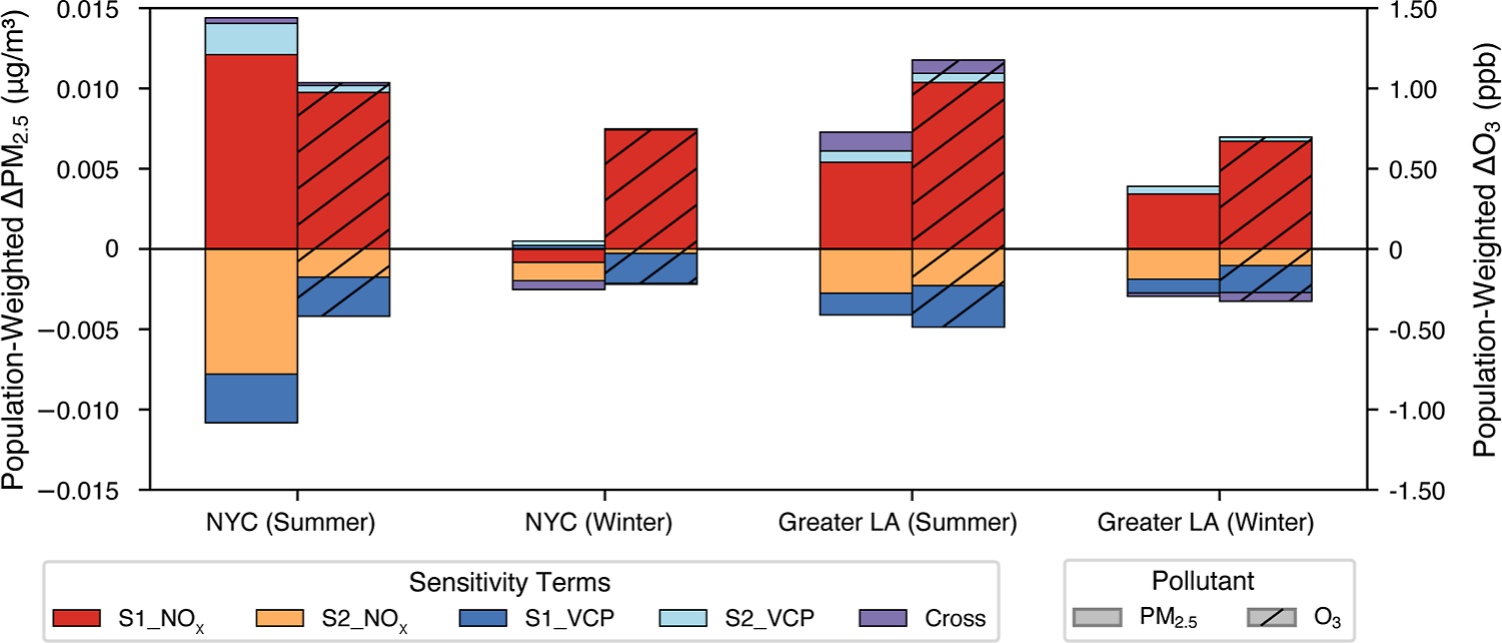
Population-weighted PM_2.5_ and O_3_ concentration changes in NYC and Greater LA attributable to each sensitivity terms for a 50% reduction in domain-wide NO_*x*_ and VCP emissions. For example, the red bars (S1_NO_*x*_) represent the impact from the first order sensitivity term (i.e., $s_{PM_{2.5}|NO_{x}}^{\left(1\right)}$ and $s_{O_{3}|NO_{x}}^{\left(1\right)}$ ) on population-weighted concentrations. O_3_ concentrations responses are indicated by hatchings on bars. Four scenarios are presented: NYC (summer), NYC (winter), Greater LA (summer), and Greater LA (winter). Positive values indicate that the emission reductions results in an increase in the population-weighted concentration of the pollutant (e.g., the hatched red bar for NYC in summer shows that reducing NO_*x*_ increases population-weighted O_3_ concentrations). The impact from the cross-sensitivity ( $s_{O_{3}|NO_{x},VCP}^{\left(2\right)}$ ) is labeled by a purple color bar.

For PM_2.5_, the impacts of NO_*x*_ and VCP emission reductions are generally small across all four scenarios, yet the derivative-based framework in CMAQ-hyd allows these marginal changes to be quantified. For example, the first-order sensitivity of PM_2.5_ to NO_*x*_ (S1_NO_*x*_) is positive in NYC (summer) and Greater LA (summer and winter), suggesting that NO_*x*_ reductions can increase oxidant levels and potentially promote secondary PM_2.5_ formation. In these cases, the second-order sensitivity (S2_NO_*x*_) is negative, partially offsetting the first-order effect. Cross sensitivities for PM_2.5_ are generally minor, except for Greater LA in summer, where the positive cross term indicates that simultaneous NO_*x*_ and VCP reductions lead to higher PM_2.5_ concentrations: an antagonistic interaction between the two precursors. VCP impacts on PM_2.5_ are consistently smaller than those of NO_*x*_; for example, in NYC (summer), the first- and second-order sensitivities to VCP reductions nearly cancel each other, illustrating the importance of accounting for higher-order terms.

For O_3_, consistent with results shown in earlier figures, NO_*x*_ reductions in densely populated urban centers including NYC and Greater LA tend to increase concentrations under VOC-limited conditions. Across all four scenarios, positive S1_NO_*x*_ and negative S2_NO_*x*_ implies a negative first-order sensitivity (higher O_3_ with lower NO_*x*_) that is partially counteracted by a positive second-order effect. The magnitude of the first-order term exceeds that of the second-order term, resulting in a net increase in population-weighted O_3_ when NO_*x*_ is reduced. For VCP, S1_VCP is negative while S2_VCP is small or slightly positive across all four scenarios, indicating that VCP reductions generally reduce O_3_ concentrations, with only minor higher-order effects. Cross sensitivities for O_3_ are small in most cases, though Greater LA in summer again shows a positive cross term, suggesting antagonistic effects of simultaneous NO_*x*_ and VCP reductions. These results demonstrate the capability of CMAQ-hyd to attribute changes in population-weighted exposure to individual and interacting precursor emissions with machine-level precision, highlighting the importance of including higher-order terms when developing control strategies for chemically complex urban environments.

## 4 Conclusion, Limitations, and Future Directions

This study evaluated the impacts of domain-wide, marginal reductions in VCP and NO_*x*_ on O_3_ and PM_2.5_ and the associated acute health benefits. However, policy approaches are rarely implemented at such comprehensive spatial scales. Air pollution control strategies are typically implemented within a region or for a sector. As demonstrated in Figure 4, the chemical regime varies significantly between urban cores and suburban downwind areas; therefore, future work should examine localized and sector-specific reduction strategies (e.g., reducing only on-road NO_*x*_ emissions).

In addition, the study was performed on a 12 km × 12 km grid, which may not capture intricate urban atmospheric interactions within densely populated urban centers. Within metropolitan areas, O_3_ and PM_2.5_ concentrations vary at the neighborhood scale, with localized hotspots often coinciding with vulnerable populations. Consequently, the coarse resolution applied in this study could not be applied to neighborhood-scale analysis of pollutant exposure. Future applications of CMAQ-hyd or similar frameworks at finer spatial scales will be critical for resolving these local variations and better informing equitable air quality management strategies.

This study also only focuses on interactions between controllable, anthropogenic emissions. Although biogenic VOC emissions are not controllable, interactions between biogenic VOC and anthropogenic NO_*x*_ can be evaluated using the augmented CMAQ-hyd model.^25^ By capturing the higher-order sensitivities of pollutants and the cross-terms between anthropogenic and biogenic precursors on O_3_ and PM_2.5_ formation, this type of modeling provides policymakers with insights into the nonlinear atmospheric chemistry driving the formation. As on-road NO_*x*_ emissions continue to decline,^44^ urban photochemical regimes are shifting and the relative importance of biogenic VOC contributions to O_3_ and secondary PM_2.5_ formation is being amplified in accordance with these nonlinear effects. Observations during COVID-19 lockdown periods further demonstrated that abrupt NO_*x*_ emission reductions from on-road vehicles can enhance O_3_ in urban areas,^23,45^ underscoring the need for advanced sensitivity frameworks capable of disentangling the coupled anthropogenic–biogenic interactions that govern these nonlinear responses. Such analyses will be especially valuable to regions with strong biogenic emission sources proximate to urban centers and could be further expanded to predict air pollution under a warming climate.

Finally, we maintained an achievable, narrow focus on the acute premature mortality effects of O_3_ and PM_2.5_.^34^ With sufficient computational resources and time, the same approach could be extended to premature mortality associated with chronic exposure to these pollutants in future work.^46,47^

Beyond concentration-based outcomes, this study demonstrated how first-, second-, and cross-sensitivities can be used to estimate impacts on acute premature mortality through Taylor expansion. CMAQ-hyd can be linked with health and economic assessment frameworks, for example, by applying the statistical value of a life to monetize exposure changes or by coupling with tools such as the AVoided Emissions and Generation Tool (AVERT) to evaluate the air quality implications of energy efficiency, renewable energy deployment, and electric vehicle adoption. These linkages highlight the versatility of sensitivity-based approaches for connecting atmospheric modeling to policy-relevant decision support.

In conclusion, this study demonstrates the value of higher-order sensitivity analysis for quantifying the linear and nonlinear effects of relatively novel precursor emissions on O_3_ (Figure 3) and PM_2.5_ (Figure S11) and for linking atmospheric modeling with health and policy evaluations. By applying CMAQ-hyd at finer spatial scales, incorporating biogenic–anthropogenic interactions, and expanding to additional precursors and health outcomes, future work can further strengthen the scientific foundation for effective air quality management.

## Supplementary Material



## Data Availability

The CMAQ-hyd model is archived at 10.5281/zenodo.7938725, and is under a MIT license.^48^ The scripts used for visualizations and analyses can be found at https://zenodo.org/records/17362555.^49^

## Notes

LiuCappsNOxVCPsi.r3 (PDF)
