# Supplementary material for: Nonlinear Contributions of NO x and Volatile Chemical Products to Air Pollution and the Associated Acute Premature Mortality
Source: ACS EST Air. 2026 May 7;3(6):1462–72. doi: 10.1021/acsestair.5c00415 (PMC13270514; doi:10.1021/acsestair.5c00415)
Supplement: Supplementary file 1 [file ea5c00415_si_001.pdf]

Nonlinear Contributions of NO_x and Volatile Chemical Products to Air Pollution and the Associated Acute Premature Mortality

Supporting Information

Jiachen Liu[1] and Shannon L. Capps[1]

[1]Department of Civil, Architectural & Environmental Engineering, Drexel University, 3141 Market St, Philadelphia, PA, 19104, USA

Corresponding author: Shannon L. Capps, shannon.capps@drexel.edu

Compromises made during the development and implementation of the CMAQ-hyd model

Several compromises have been made to stabilize the sensitivity coefficients calculated by CMAQ-hyd. In our previous paper[1], we discussed compromises made including the implementation of a temperature and pressure threshold for forward ISORROPIA. Additional compromises for the continental-scale sensitivity analysis include the following:

1) The sensitivities from a specific reverse ISORROPIA subroutine, CALCL9, are ignored. No sensitivities from this specific subroutine contributes to the overall sensitivity of the model.

2) The sensitivities from the aqueous chemistry in clouds are ignored due to potential numerical instability. Similar computational methods including the direct decoupled method (CMAQ-DDM) also made compromises in the cloud module to avoid computational instability.

3) The sensitivities from the photolysis module are ignored because they will minimally affect the concentration to emission relationship.

Discussion on modeling representativeness (Figures S3–S6)

To ensure the selected one-week modeling episodes are representative of average summer and winter conditions, we compared episode-average concentrations (July 16–22 for summer; January 16–22 for winter) against the corresponding seasonal averages (JJA and DJF) from EQUATES at every CONUS grid cell (Figure S3). For the summer episode, spatial correlations are strong across the domain for both O_3 (R^2=0.868) and $PM_{2.5}$ (R^2=0.575), with regression slopes near unity, indicating that the episode captures both the magnitude and spatial pattern of seasonal concentrations. For the winter episode, $PM_{2.5}$ shows good agreement (R^2=0.688), while O_3 concentrations match well in domain-average magnitude despite slightly dampened spatial gradients.

To further validate this representativeness at the urban scale where we discuss in detail in the manuscript, we evaluated the selected regional hotspots (Figure S4). Both the New York City (NYC) and Greater Los Angeles (LA) domains demonstrate robust concentration agreement across the grid cells, with combined summer O_3exhibiting an R^2 of 0.903 and winter $PM_{2.5}$ showing an R^2 of 0.862.

Because the primary output of our analysis is the sensitivity of health impacts to precursor emission changes. We also evaluated the consistency of the O_3/NO_x ratio between the episode and the seasonal average. Across CONUS (Figure S5), the episode and the seasonal average agree with each other (R^2=0.951 for summer; R^2=0.882 for winter). At the local scale (Figure S6), this chemical consistency is strongly maintained. The O_3/NO_x ratios between the episode and seasonal averages show excellent correlation in both the purely VOC-limited urban NYC domain (R^2=0.912 for summer; R^2=0.908 for winter) and the larger LA domain (R^2=0.946 for summer; R^2=0.790 for winter), which contains urban, suburban, and rural scenarios.

In conclusion, these results demonstrate that the nonlinear concentration response surfaces during the modeling period could be applicable to evaluate seasonal conditions, as the underlying local chemistry governing concentration-emission relationships is preserved.

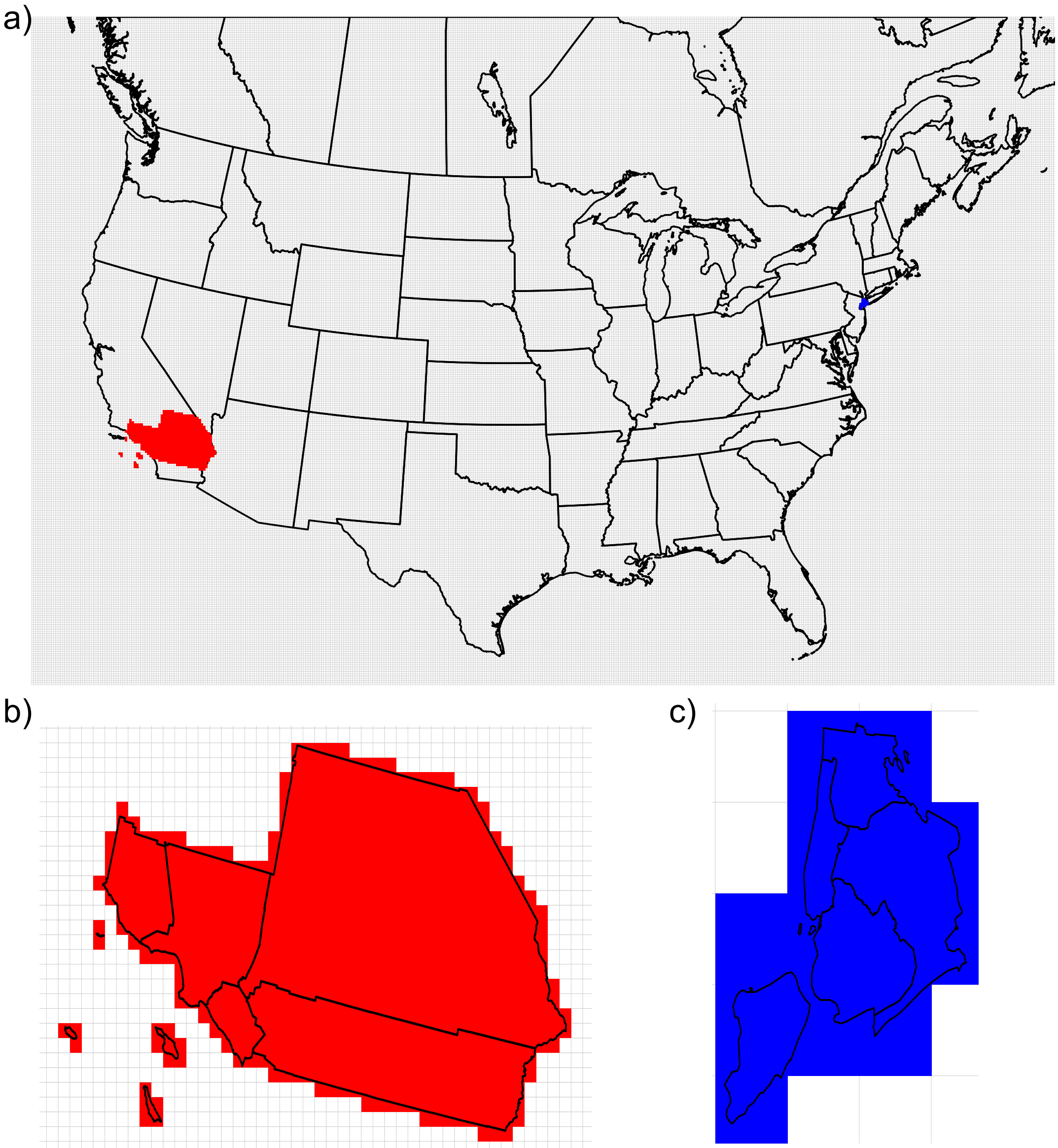

Figure S1. The full CMAQ-hyd modeling domain a) consisting of 499 x 259 grid cells across continental United States. The The Greater Los Angeles (LA) and (c) New York City (NYC) subdomains are highlighted on the full domain. The Greater LA region includes Los Angeles, Orange, Ventura, San Bernardino, and Riverside Counties, while the NYC domain comprises New York, Kings, Bronx, Richmond, and Queens Counties.

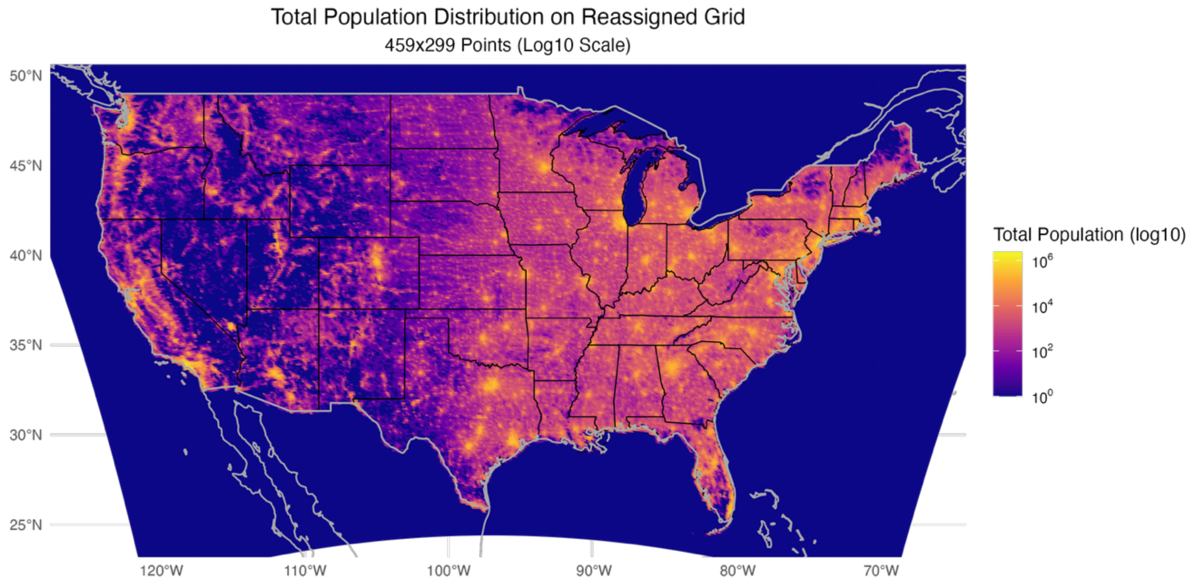

Figure S2. The population map for the modeling domain. The population data is derived from 2019 U.S. Census Bureau population estimates and regridded from the 12US2 grid to 12US1 grid.

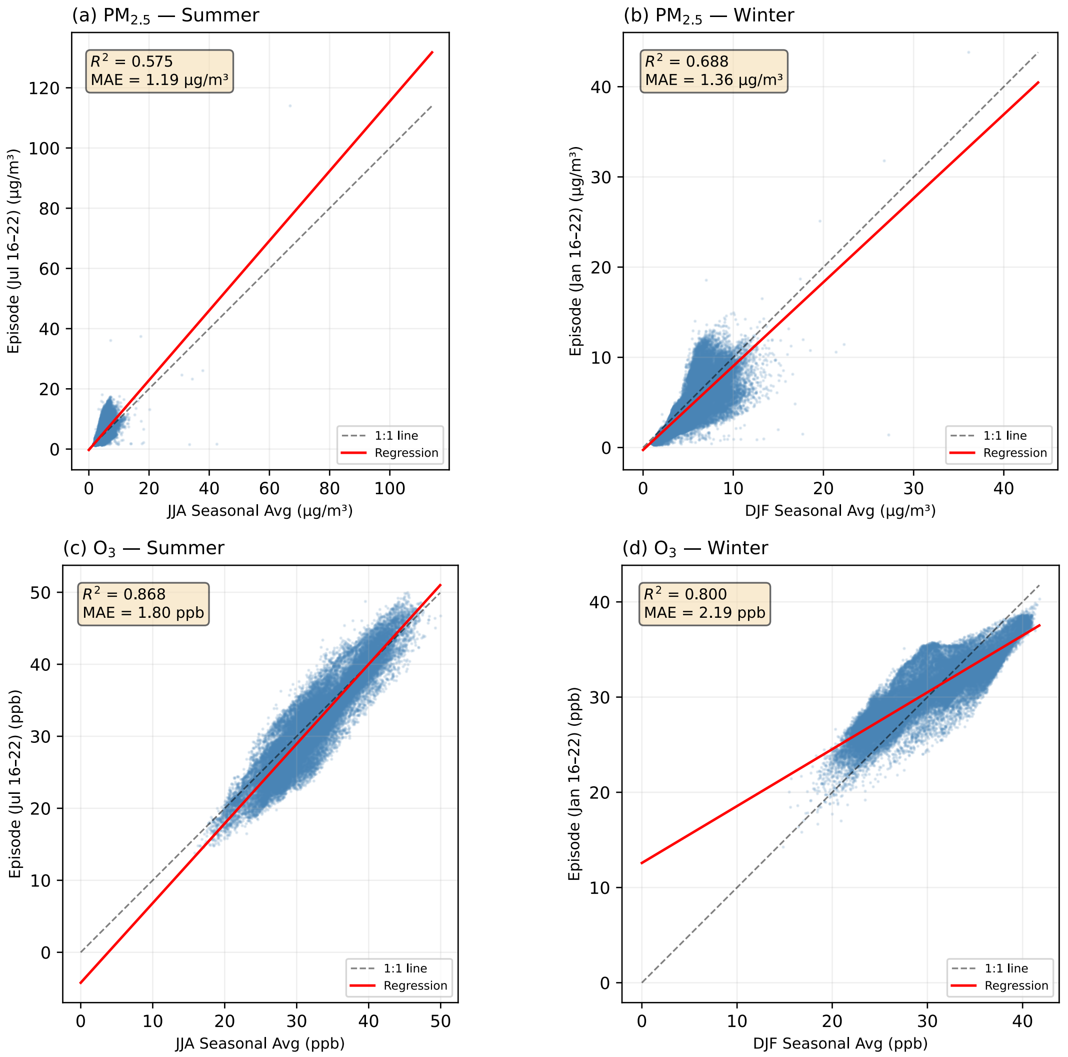

Figure S3. Comparison of episode-average concentrations against corresponding EQUATES seasonal averages for $PM_{2.5}$ (a, b) and O_3 (c, d) across all contiguous United States (CONUS) grid cells. Summer comparisons (July 16–22 vs. JJA) are shown on the left, and winter comparisons (January 16–22 vs. DJF) on the right. Solid red lines indicate the linear regression, and dashed grey lines represent the 1:1 line.

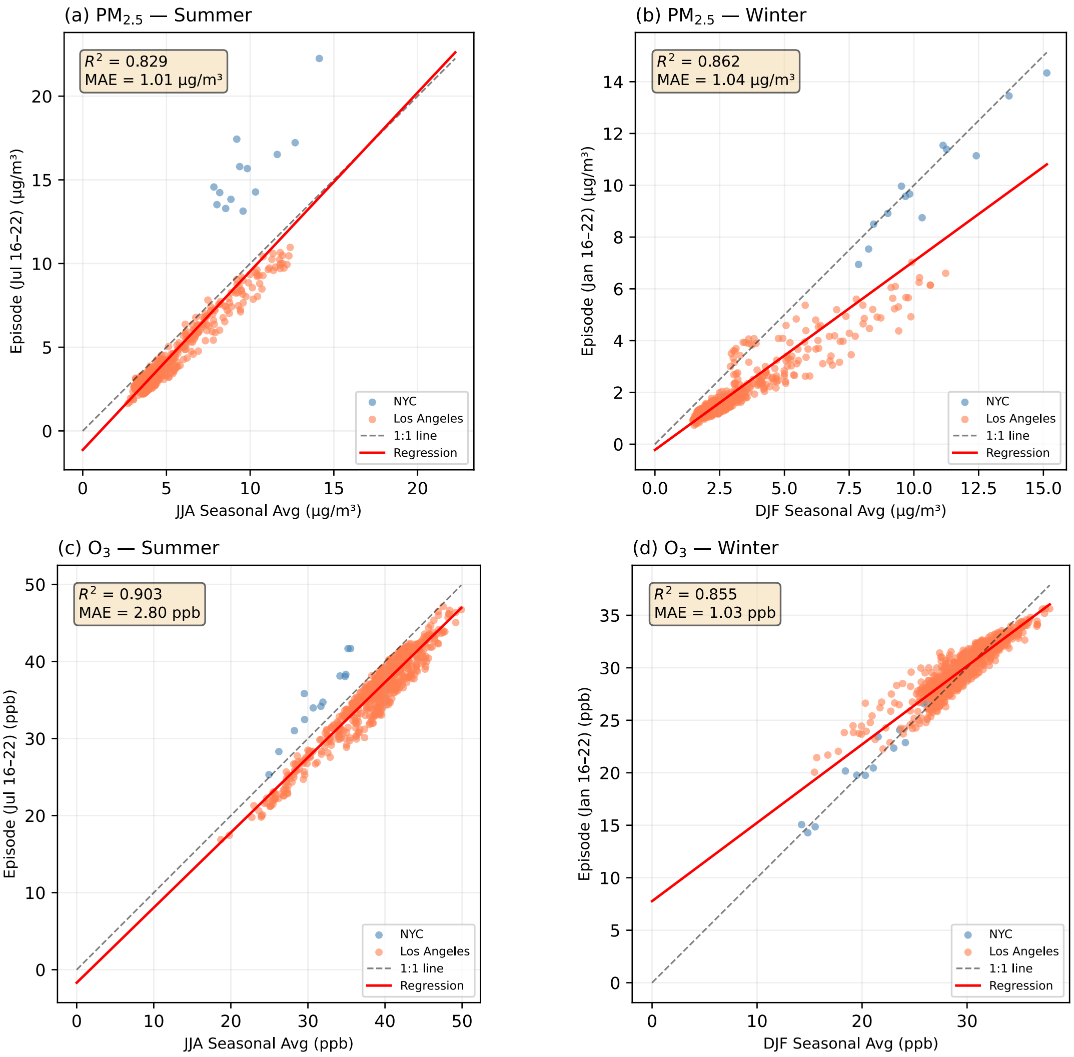

Figure S4. Comparison of episode-average concentrations against corresponding EQUATES seasonal averages for $PM_{2.5}$ (a, b) and O_3 (c, d) within the NYC (blue points) and greater LA (orange points) domains. Summer comparisons (July 16–22 vs. JJA) are shown on the left, and winter comparisons (January 16–22 vs. DJF) on the right. Solid red lines indicate the linear regression, and dashed grey lines represent the 1:1 line.

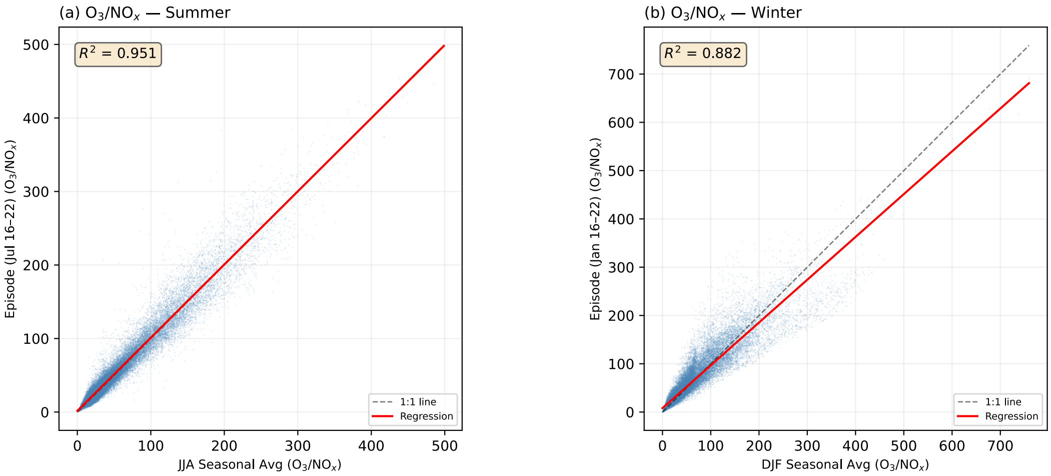

Figure S5. Comparison of the O_3/NO_x ratio between the selected modeling episodes and seasonal averages for the CONUS domain. Summer comparisons are shown on the left, and winter comparisons on the right. Solid red lines indicate the linear regression, and dashed grey lines represent the 1:1 line.

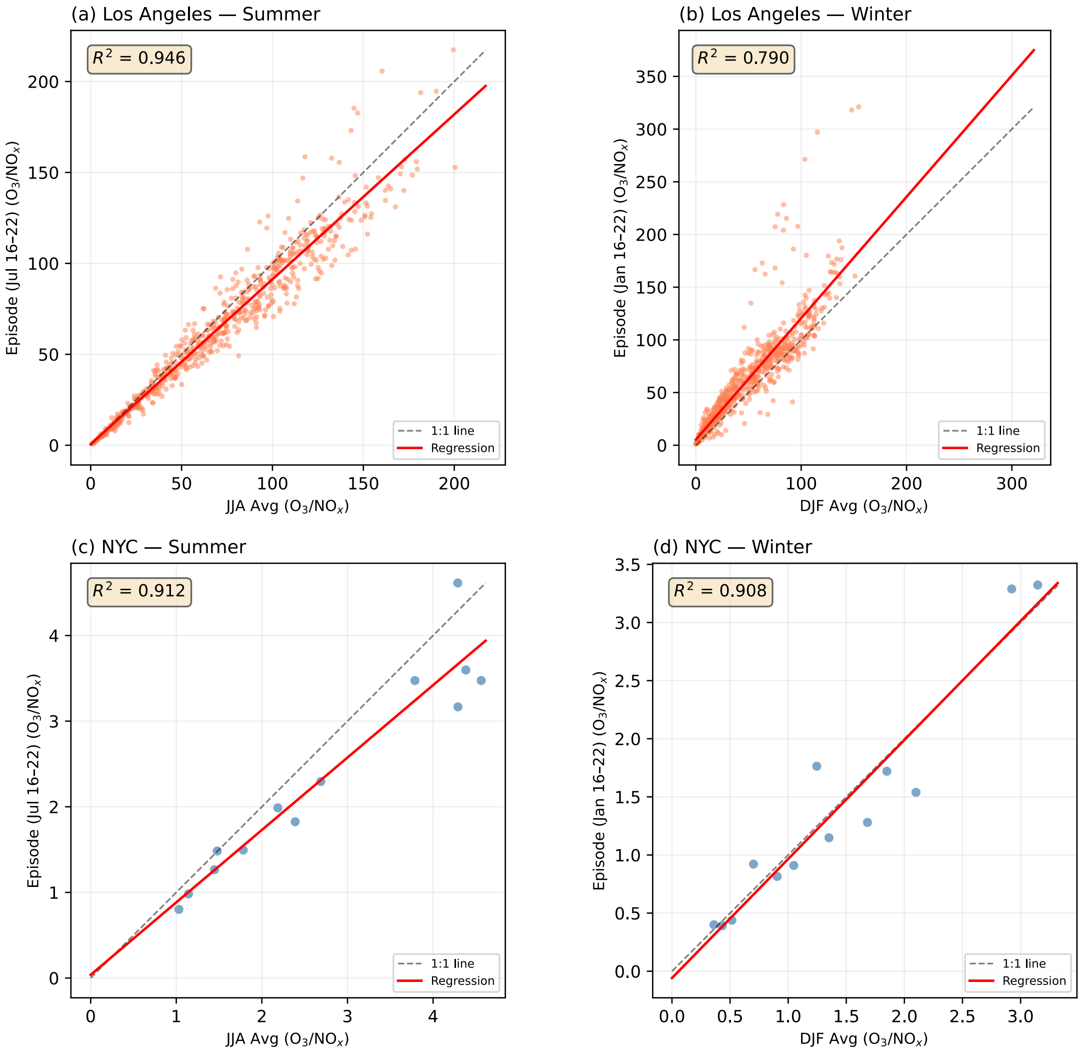

Figure S6. Comparison of the O_3/NO_x ratio between the selected modeling episodes and seasonal averages for the Greater LA (a, b) and NYC (c, d) domains. Summer comparisons are shown on the left, and winter comparisons on the right. Solid red lines indicate the linear regression, and dashed grey lines represent the 1:1 line.

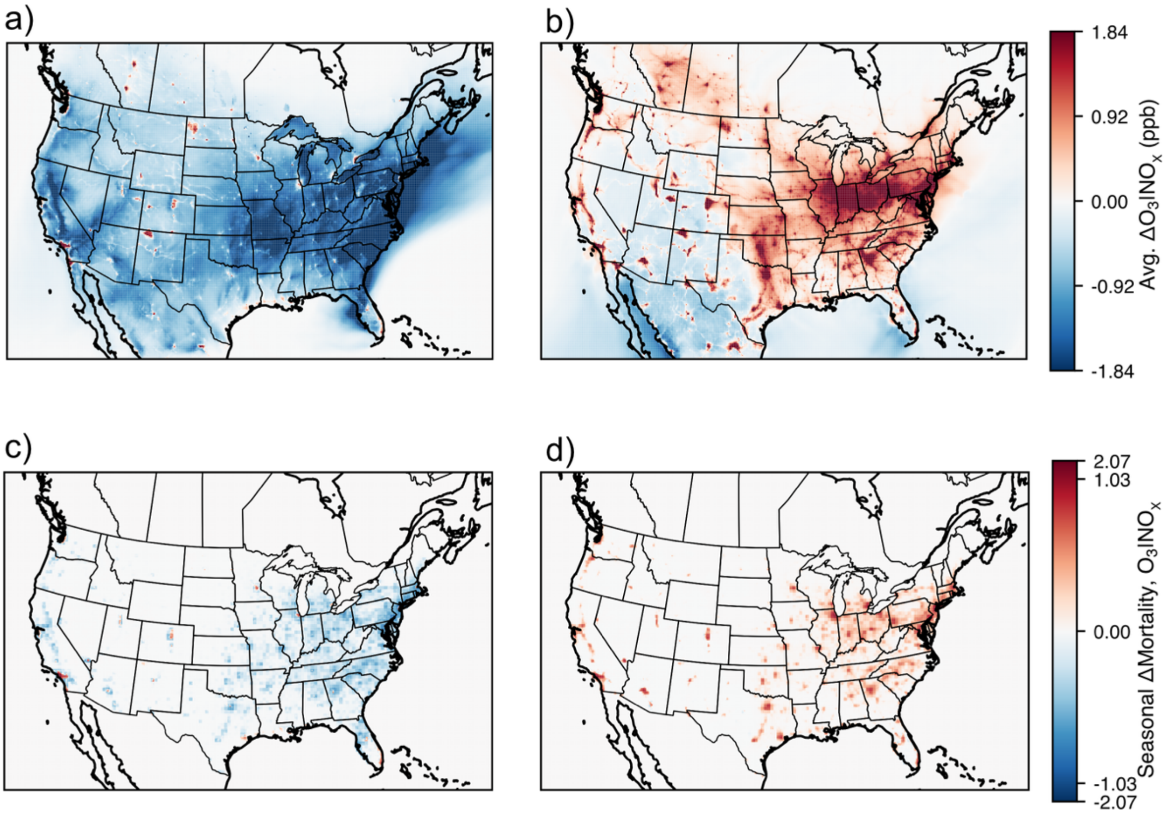

Figure S7. The O_3 concentration changes due to a 50% reduction in domain-wide reduction in NO_x emissions in summer a) and in winter b). The changes in mortality due to O_3 in summer c) and in winter d). Concentration changes panels use a linear, two-slope color scale clipped at the ±98th percentile of the delta-values to avoid plotting the extreme values on the colormap.

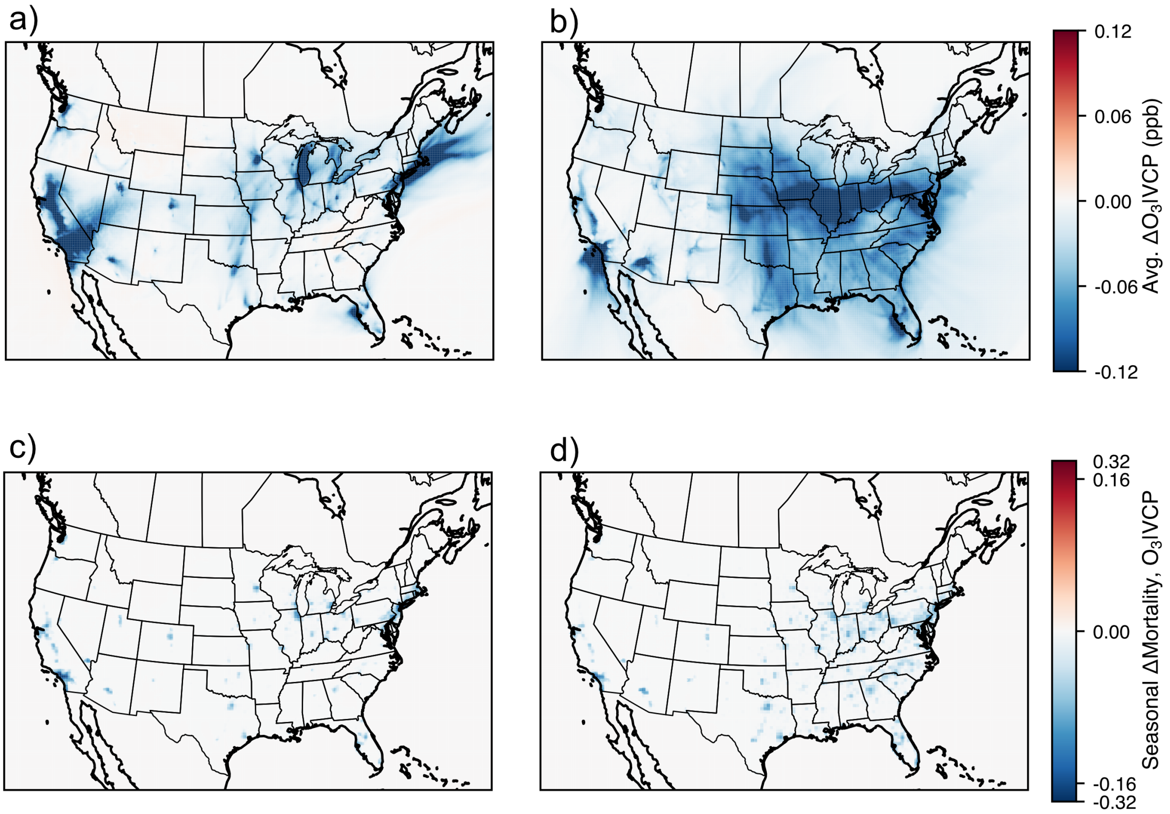

Figure S8. The O_3 concentration changes due to a 50% reduction in domain-wide reduction in VCP emissions in summer a) and in winter b). The changes in mortality due to O_3 in summer c) and in winter d). Concentration changes panels use a linear, two-slope color scale clipped at the ±98th percentile of the delta-values to avoid plotting the extreme values on the colormap.

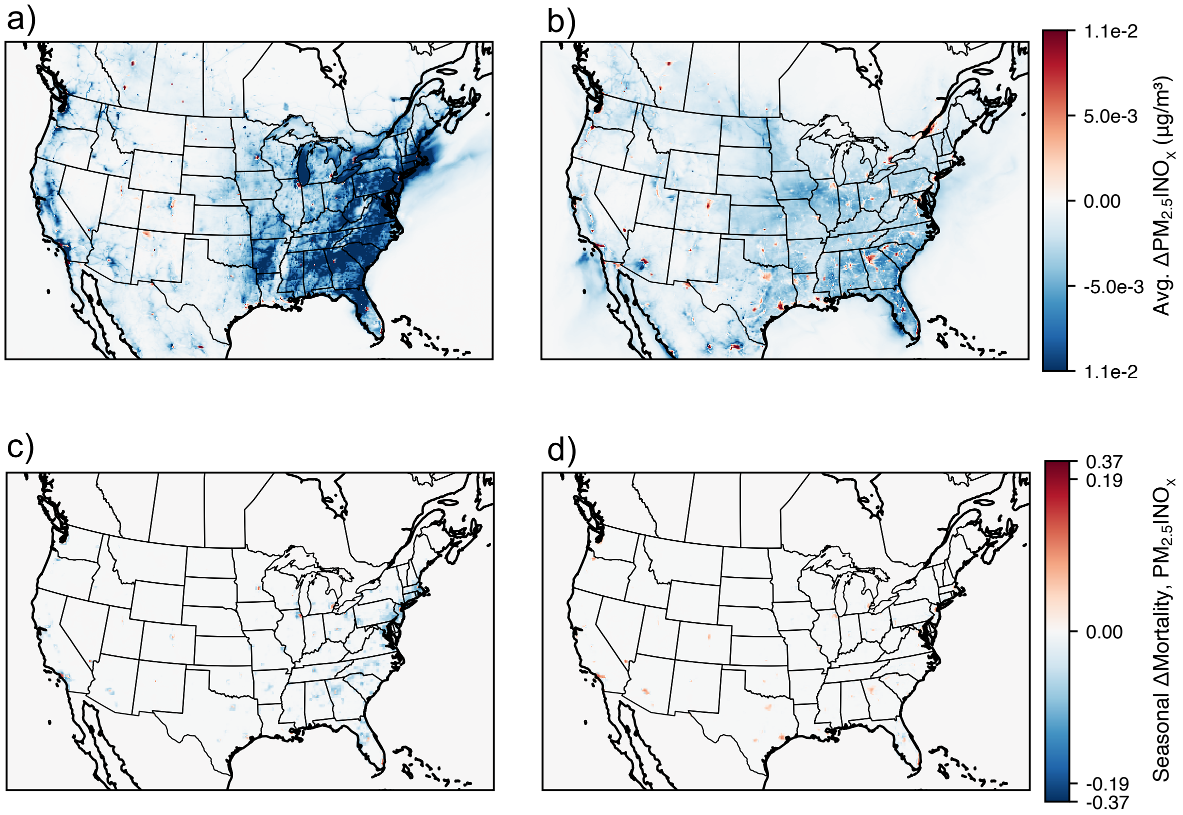

Figure S9. The $PM_{2.5}$ concentration changes due to a 50% reduction in domain-wide reduction in NO_x emissions in summer a) and in winter b). The changes in mortality due to $PM_{2.5}$ in summer c) and in winter d). Concentration changes panels use a linear, two-slope color scale clipped at the ±98th percentile of the delta-values to avoid plotting the extreme values on the colormap.

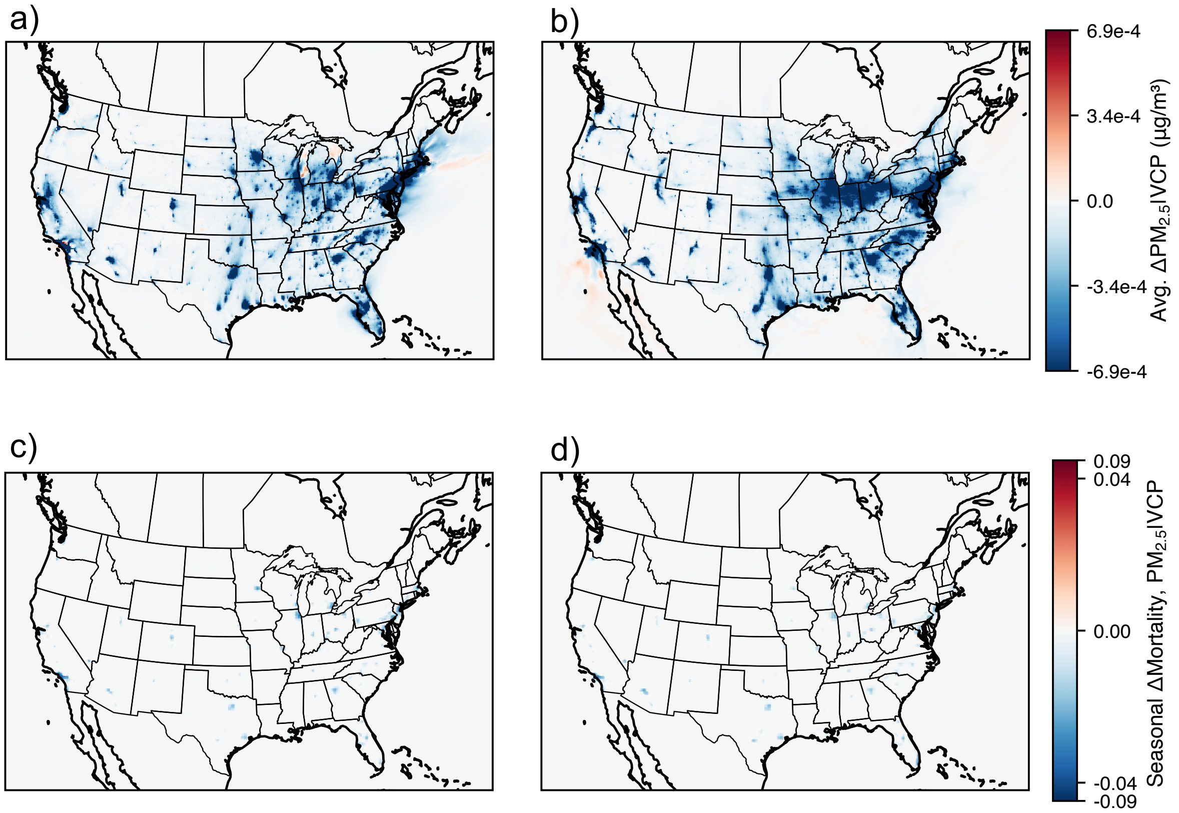

Figure S10. The $PM_{2.5}$ concentration changes due to a 50% reduction in domain-wide reduction in VCP emissions in summer a) and in winter b). The changes in mortality due to $PM_{2.5}$ in summer c) and in winter d). Concentration changes panels use a linear, two-slope color scale clipped at the ±98th percentile of the delta-values to avoid plotting the extreme values on the colormap.

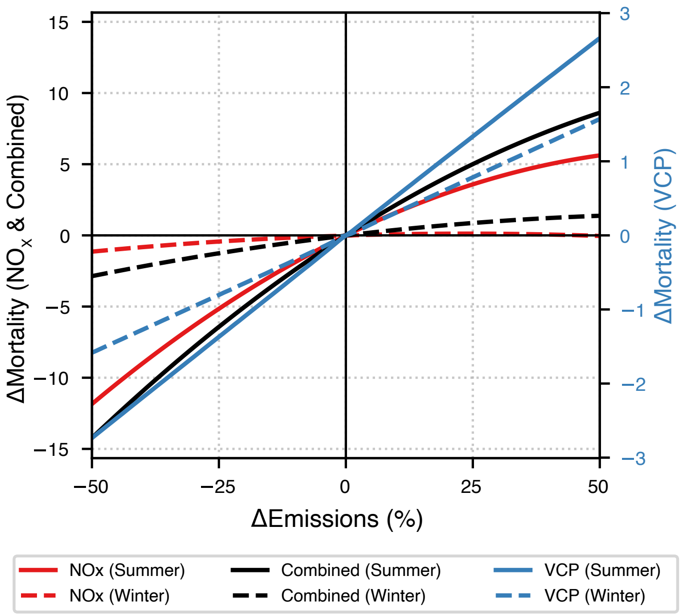

Figure S11. Cumulative changes in mortality from $PM_{2.5}$ due to NO_x (red), VCP (blue), and combined impact of both (black) in summer (solid line) and in winter (dashed line) with the range of change in emissions from -50% to +50%. Two separate *y*-axes are employed to demonstrate the impact of NO_x and combined impact (black *y*-axis) and the impact of VCP (blue *y*-axis)

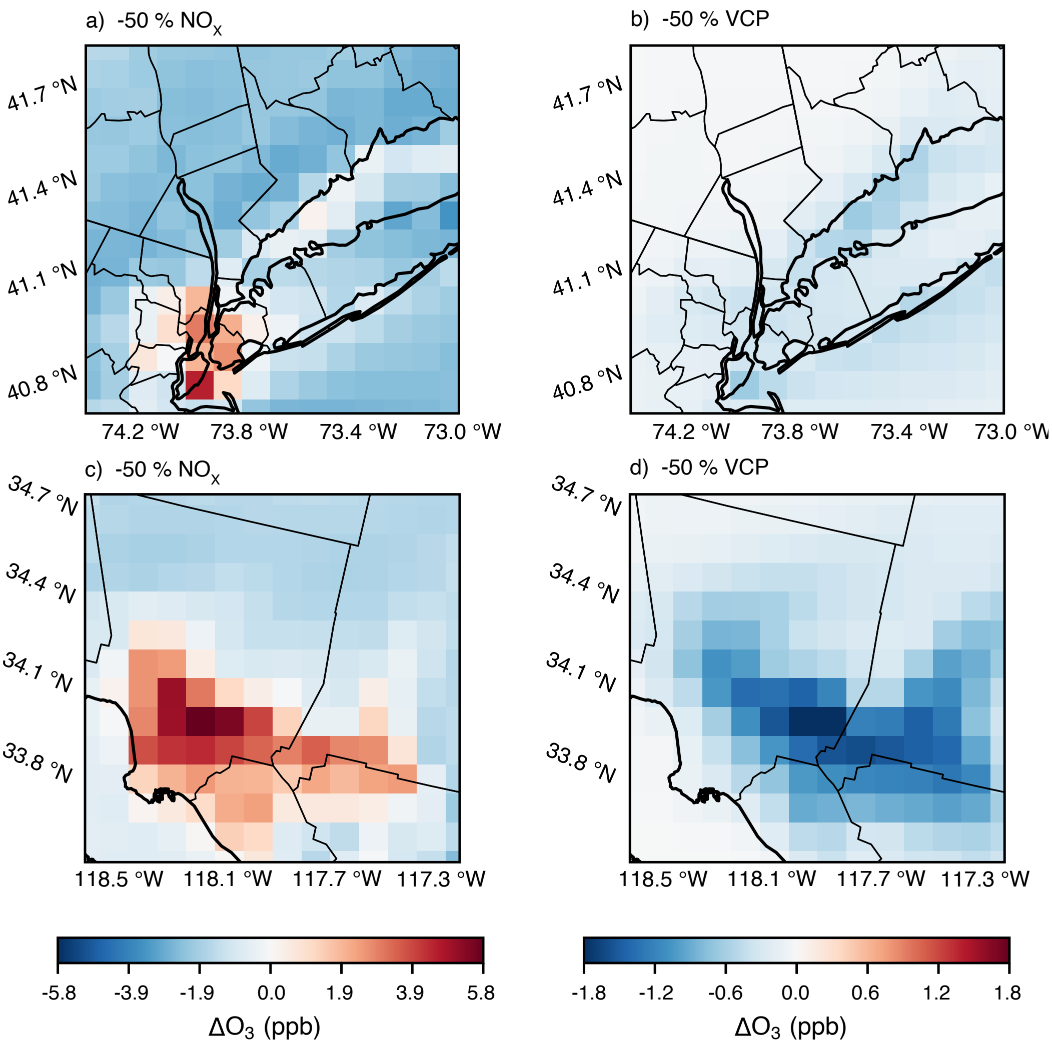

Figure S12. The spatial distribution of changes in O_3 concentrations (ΔO_3) within these domains resulting from a 50% reduction NO_x and VCP emissions. Left panels (a, c) illustrate the O_3 response to a 50% reduction in NO_x emissions for NYC grid cells (top) and LA grid cells (bottom), while right panels (b, d) illustrate the response to a 50% reduction in VCP emissions for the NYC grid cells (top) and LA grid cells (bottom) domains.

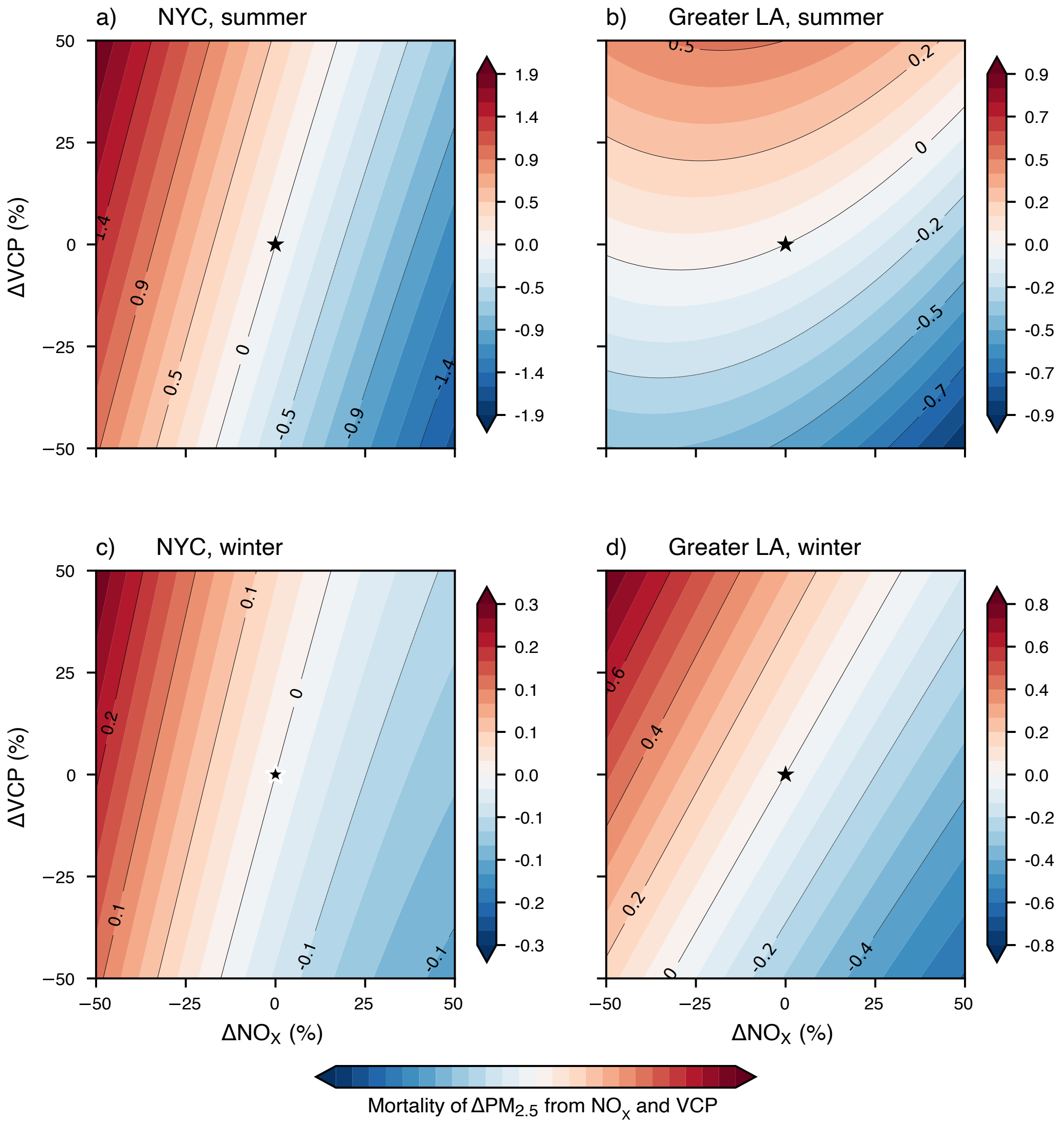

Figure S13. Total $PM_{2.5}$-related mortality changes in response to domain-wide NO_x and VCP emission perturbations, calculated using Equation (5) and aggregated for four urban-region and seasonal scenarios: (a) NYC in summer, (b) Greater LA in summer, (c) NYC in winter, and (d) Greater LA in winter. Emission perturbations for both NO_x and VCPs range from −50% to +50%. All mortality estimates account for both first-order and cross-sensitivities between precursors.

Table S1. Domain-wide total seasonal changes in mortality from 10% reduction in emissions. The 95% confidence intervals (CIs) are derived from the uncertainty in the concentration–response coefficient (β).

Season	Emission	Pollutant	ΔMortality (95% CI)
Summer	NO_x	$PM_{2.5}$	-1.9 [-1.4, -2.4]
		O_3	-59.1 [-30.7, -87.4]
	VCP	$PM_{2.5}$	-0.55 [-0.40, -0.69]
		O_3	-5.6 [-2.9, -8.2]
	Combined	$PM_{2.5}$	-2.4 [-1.7, -3.0]
		O_3	-373.2 [-194.0, -552.0]
Winter	NO_x	$PM_{2.5}$	-0.15 [-0.11, -0.18]
		O_3	102.5 [53.3, 151.5]
	VCP	$PM_{2.5}$	-0.33 [-0.24, -0.41]
		O_3	-5.2 [-2.7, -7.8]
	Combined	$PM_{2.5}$	-0.47 [-0.34, -0.58]
		O_3	97.2 [50.5, 143.8]

Table S2. Domain-wide total seasonal changes in mortality from 25% reduction in emissions. The 95% confidence intervals (CIs) are derived from the uncertainty in the concentration–response coefficient (β).

Season	Emission	Pollutant	ΔMortality (95% CI)
Summer	NO_x	$PM_{2.5}$	-5.2 [-3.7, -6.4]
		O_3	-158.5 [-82.4, -234.4]
	VCP	$PM_{2.5}$	-1.4 [-1.0, -1.7]
		O_3	-14.0 [-7.3, -20.7]
	Combined	$PM_{2.5}$	-6.4 [-4.6, -8.0]
		O_3	-170.5 [-88.6, -252.1]
Winter	NO_x	$PM_{2.5}$	-0.43 [-0.31, -0.54]
		O_3	256.9 [133.6, 379.8]
	VCP	$PM_{2.5}$	-0.80 [-0.58, -0.99]
		O_3	-13.1 [-6.8, -19.3]
	Combined	$PM_{2.5}$	-1.3 [-0.9, -1.6]
		O_3	243.6 [126.7, 360.2]

References

(1) Liu, J.; Chen, E.; Capps, S. L. The First Application of a Numerically Exact, Higher-Order Sensitivity Analysis Approach for Atmospheric Modelling: Implementation of the Hyperdual-Step Method in the Community Multiscale Air Quality Model (CMAQ) Version 5.3.2. *Geosci. Model Dev.* **2024**, *17* (2), 567–585. https://doi.org/10.5194/gmd-17-567-2024.
